# Soils of the Southern Syria – A big database for the future land management planning

**DOI:** 10.1016/j.dib.2020.105832

**Published:** 2020-06-11

**Authors:** Safwan Mohammed, Hassan Habib, Haidar Ali, Sami AlHennaw, Samer Kiwan, Samar Ghanem, Karam Alsafadi, Eric C. Brevik, Magboul M. Sulieman, Endre Harsányi

**Affiliations:** a Institute of Land Use, Technology and Regional Development, Faculty of Agricultural and Food Sciences and Environmental Management, University of Debrecen, Debrecen 4032, Hungary; b Faculty of Agricultural, Department of Soil Science, Damascus University, Damascus, Syria; c Administration of Natural Resources Management, General Commission for Scientific Agricultural Research, Damascus, Syria; d Department of soil and water science, Faculty of Agriculture, Tishreen University, Lattakia, Syria; e Department of Geography and GIS, Faculty of Arts, Alexandria University, Alexandria 25435, Egypt; f Department of Natural Sciences, Dickinson State University, Dickinson, ND, USA; g Department of Agriculture and Technical Studies, Dickinson State University, Dickinson, ND, USA; h Department of Soil and Environment Sciences, Faculty of Agriculture, University of Khartoum, Khartoum North, 13314 Shambat, Sudan; i Soil Sciences Department, College of Food and Agricultural Sciences, King Saud University, P.O. Box 2460, Riyadh 11451, Saudi Arabia

**Keywords:** Palygorskite, Rehabilitation, Soil classification, Southern Syria

## Abstract

As non-renewable natural resources, restoring Syrian soil quality is a vital issue for sustainable future planning after conflict ends. The data provided in this research exhibit features and physiochemical properties for soils from the southern part of Syria until the Jordanian border, which can provide decision-makers with sufficient information for rehabilitation stage after conflict in a regional scale. The data were collected from 107 representative soil profiles covering diverse agroecosystems throughout the area (i.e. Dara and Alswieda governorates). The most important data findings of this research included the first detection of *Palygorskite* {(Mg,Al)_2_Si_4_O_10_(OH)•4(H_2_O)} in Syrian soils, which is considered a strong evidence for the direct effects of the climate change on agroecosystem. Vertisols, Inceptisols, Entisols, Mollisols, and Aridisols were the most widespread soil types in the area. Overall, the database involves the field morphological characteristics, physicochemical, and mineralogical analyses.

Specifications table

**Table:** 

Subject	Soil science
Specific subject area	Soil classification
Type of data	Table, image
How data were acquired	Soil samples and morphological data were collected according to the FAO guidelines for soil profile description between 2013–2016. Soil sample analyses were carried out using standard soil methods. Soil mineralogical data were acquired using X-ray powder diffraction (XRD) technique.
Data format	Raw, analysed
Parameters for data collection	Soil samples were air- dried and were passed through a 2 mm sieve for list of chemical and physical analyses.
Description of data collection	For deep soil characteristics investigation in the study area, 107 soil profiles were digging till the parent material. Accordingly, soil samples were collected from each horizon. Routine soil analyses were carried out for physicochemical properties using standard soil laboratory methods. Selected soil samples were analysed for mineralogical composition using XRD.
	From the southern part of Syria (Dara and Alswieda governorates), the rest coordinates for the studied sites are given in Table 1:
Data source location	SYSD01 36.70416667 E 32.69583333 N
	SYSW02 36.6015 E 32.75239 N
	SYSW13 36.55764 E 32.74922 N
	SYSW31 36.45254 E 32.70306 N
	SYSW35 36.35778 E 32.7587 N
	SYD09 36.129444 E 32.830555 N
	SYD12 36.1730555 E 32.833611 N
	SYD26 36.10916667 E 32.7152778 N
	SYD46 36.1744444 E 32.5344444 N
Data accessibility	The dataset is given in this data article.

**Value of the Data**

• First record of Palygorskite in southern part of Syria, which is a crucial evidence of shifting climate toward dryness, where the climate change (i.e. drought) affected soil formation and led to Palygorskite.
• Dataset provides a full overview of soil characteristics in the southern part of Syria.
• Data can be used for calibration international models in a regional scale regarding carbon sinking under ongoing climate change.
• Due to data scarcity about soil developments and classification under local Syrian conditions, this dataset will provide the scientific community with full overview of the common soil characteristics in Syria.
• Data set can provide decision-makers with detailed soil quality indicators for appropriate land use management.
• Data can be used by international organization such as FAO, ICARDA, ACSAD as a baseline for any future project regarding sustainability of land resources.

## 1 Data description

**Fig. 1:**
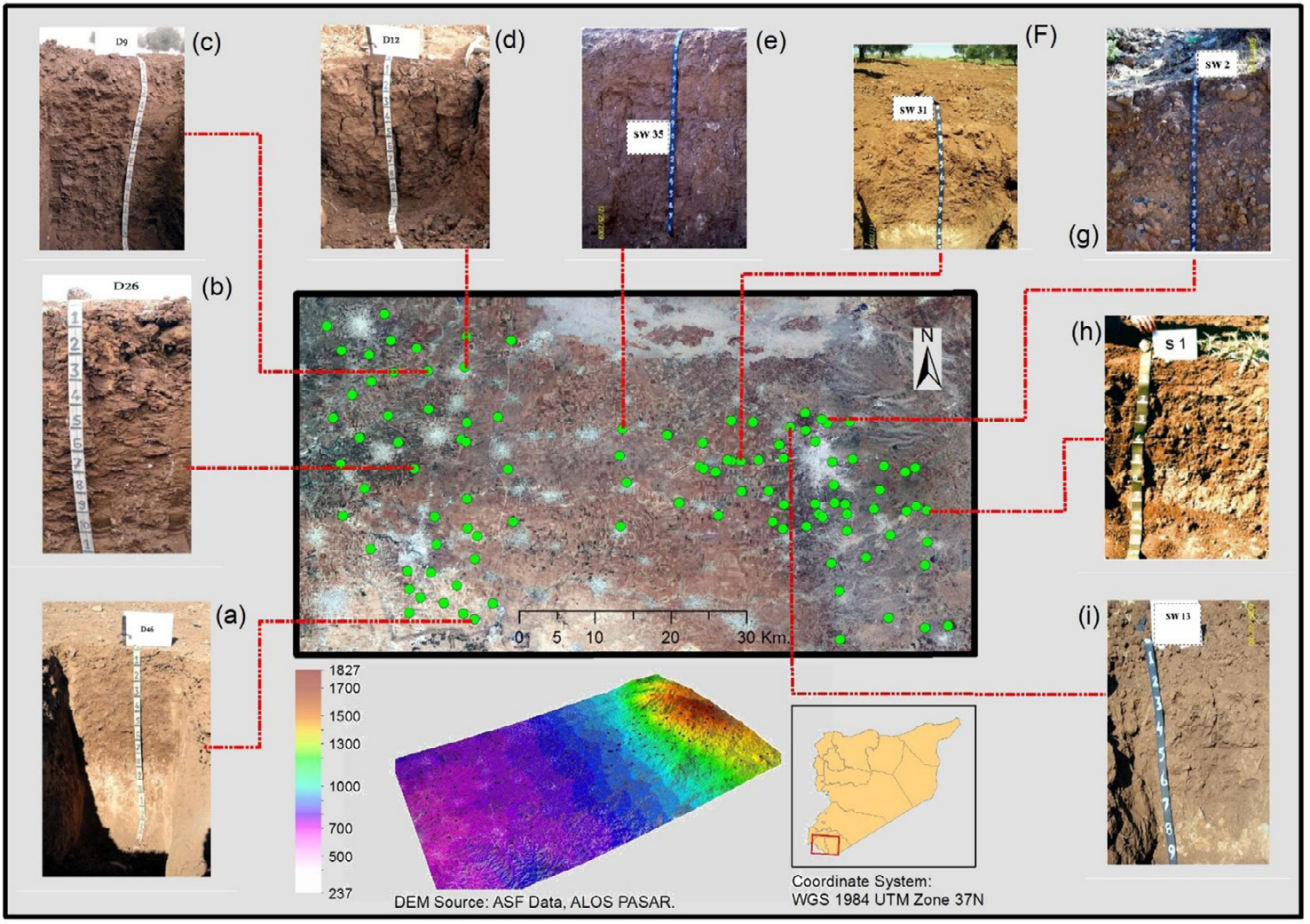
Study area and digital elevation model (DEM) maps showing the locations of selected representative soil profiles: Typic Haplocalcids (a), Chromic Calcixererts (b), Typic Haploxerepts (c), Typic Haploxererts (d), Chromic Haploxererts (e), Vertic Calcixerepts (f), Typic Haploxerolls (g), Lithic Haploxerolls (h), and Chromic Haploxererts (i).

**Fig. 2:**
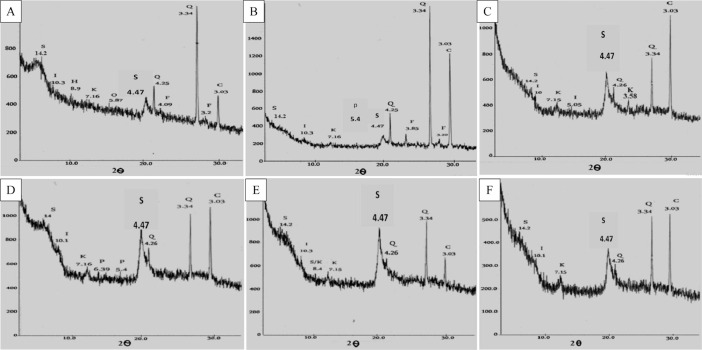
XRD patterns for selected samples from (A) SYD13 (soil sample), (B) SYD47 (soil sample), (C) SYD20 (clay sample), (D) SYD47 (clay sample), (E) SYD20 (clay sample), and (F) SYD30 (clay sample). Minerals symbols; S: Smectite, I: Illite, H: Hornblende, K: Kaolinite, O: Olivine, Q: Quartz, F: Feldspars, C: Calcite, and P: Palygorskite.

Fig. 1 shows the study area and DEM maps, and selected representative soil profiles. Fig. 2 shows the sub soil mineralogy as well as clay for some selected profiles near to Syrian and Jordanian border. Table 1 provides site properties for the selected representative soil profiles with their classification. Table 2 summarizes the field morphological descriptions for soil profiles. Table 3 presents physiochemical soil properties for soil profiles. Table 4 gives micro and macro nutrients in some representative soil profiles. Table 5 depicts the Cd and Pb heavy metals (HMs) concentrations in some representative soil profiles.

**Table 1:** Site description and soil classification for the 107 representative profiles in the southern part of Syria.

Profile	Coordinates (E/N)		Elevation (m)	Rainfall (mm)	Slope (%)	Parent material	Land cover	Land use	Soil classification*
SYDJ1	36.7042	32.6958	1650	500	Flat	Basalt	Apple trees (8 years)	Agricultural lands	Typic Haploxerolls
SYDJ2	36.6917	32.6911	1610	500	Flat	Basalt	Apple trees (12 years)	Agricultural lands	Lithic Haploxerolls
SYDJ3	36.6667	32.6992	1500	470	Flat	Basalt	Almond tree (1 year)	Agricultural lands	Typic Haploxerolls
SYDJ4	36.6608	32.6708	1500	470	Flat	Basalt	Apple trees (25 years)	Agricultural lands	Typic Haploxerolls
SYDJ5	36.63	32.7089	1340	400	10	Basalt	Annual plants	Range lands	Lithic Xerorthents
SYSW1	36.6284	32.7534	1267	400–450	2–5	Basalt⁎⁎	Apple trees+ Annual plants	Agricultural lands	Typic Haploxerolls
SYSW2	36.6015	32.7524	1164	400–450	5–10	Basalt⁎⁎	Oak trees	Forest land	Typic Haploxerolls
SYSW3	36.595	32.757	1117	300–350	2–5	Basalt⁎⁎	Vineyard+ Annual plants	Mixed lands	Typic Haploxerolls
SYSW4	36.5864	32.7307	1120	400–450	5–10	Basalt⁎⁎	Oak trees	Forest land	Typic Haploxerepts
SYSW5	36.6057	32.7064	1221	350–400	5	Basalt⁎⁎	Olive trees+ Annual plants	Mixed lands	Typic Xerorthents
SYSW6	36.6062	32.6785	1175	300–350	0.5–2	Basalt⁎⁎	Olive trees+ Annual plants	Agricultural lands	Typic Haploxerolls
SYSW7	36.6196	32.655	1262	350–400	2–5	Basalt⁎⁎	Vineyards (5 years)	Agricultural lands	Typic Haploxerolls
SYSW8	36.6211	32.6438	1284	350–400	5–10	Basalt⁎⁎	Oak trees	Forest land	Typic Haploxerolls
SYSW9	36.6069	32.6572	1197	300–350	2–5	Basalt⁎⁎	Annual crops	Agricultural lands	Humic Haploxerepts
SYSW10	36.588	32.6448	1094	300–350	0.5–2	Basalt⁎⁎	Vineyard (7 years)	Agricultural lands	Typic Haploxerepts
SYSW11	36.5755	32.7656	1027	300–350	0–0.5	Basalt⁎⁎	Vineyard (7 years)	Agricultural lands	Typic Haploxerolls
SYSW12	36.5751	32.7444	1023	300–350	5–10	Basalt⁎⁎	Oak trees	Forest land	Typic Haploxerepts
SYSW13	36.5576	32.7492	984	270–300	2–5	Basalt⁎⁎	Olive trees+ Annual crops	Agricultural lands	Chromic Haploxererts
SYSW14	36.5433	32.7288	953	270–300	2–5	Basalt⁎⁎	Annual crops	Agricultural lands	Chromic Haploxererts
SYSW15	36.5478	32.7128	965	300	2–5	Basalt⁎⁎	Annual crops	Agricultural lands	Typic Haploxerepts
SYSW16	36.529	32.6742	916	270–300	0.5–2	Basalt⁎⁎	Almond tree (7 year)	Agricultural lands	Chromic Haploxererts
SYSW17	36.5836	32.6571	1058	300	2–5	Basalt⁎⁎	Olive trees (12 years)	Agricultural lands	Typic Haploxerepts
SYSW18	36.5464	32.6572	967	300–250	0.5–2	Basalt⁎⁎	Olive trees (12 years)	Agricultural lands	Chromic Haploxererts
SYSW19	36.5329	32.6375	942	300–250	0.5–2	Basalt⁎⁎	Annual crops (Chickpea)	Agricultural lands	Vertic Haploxererts
SYSW20	36.5444	32.6288	963	300–250	0.5–2	Basalt⁎⁎	Olive trees (12 years)	Agricultural lands	Vertic Haploxererts
SYSW21	36.513	32.7568	848	300–270	2–5	Basalt⁎⁎	Annual crops	Agricultural lands	Chromic Haploxererts
SYSW22	36.486	32.7115	807	300–250	0.5–2	Basalt⁎⁎	Olive trees (2 −3 years)	Agricultural lands	Chromic Haploxererts
SYSW23	36.4877	32.759	812	270–200	0.5–2	Basalt⁎⁎	Olive trees (2 −3 years)	Agricultural lands	Calcic Haploxererts
SYSW24	36.4814	32.7128	795	275	0–0.5	Basalt⁎⁎	Olive trees (20 years)	Agricultural lands	Typic Haploxerepts
SYSW25	36.5183	32.7115	889	275	0.5–2	Basalt⁎⁎	Olive trees (12 years)	Agricultural lands	Vertic Haploxererts
SYSW26	36.4964	32.6748	872	270–200	0.5–2	Basalt⁎⁎	Olive trees (8 years)	Agricultural lands	Chromic Haploxerepts
SYSW27	36.4971	32.7104	834	275	0.5–2	Basalt⁎⁎	Olive trees (20 years)	Agricultural lands	Typic Haploxerepts
SYSW28	36.4528	32.7342	806	275	0.5–2	Basalt**	Olive trees (15 years)	Agricultural lands	Chromic Haploxererts
SYSW29	36.4485	32.7064	762	270–200	0.5–2	Basalt⁎⁎	Olive trees (10 years)	Agricultural lands	Vertic Haploxerepts
SYSW30	36.411	32.745	732	275	2–5	Basalt⁎⁎	Annual crops	Agricultural lands	Chromic Haploxererts
SYSW31	36.4525	32.7031	815	270	2	Basalt⁎⁎	Olive trees (15 years)	Agricultural lands	Vertic Calcixerertps
SYSW32	36.4686	32.6472	785	270	1	Basalt⁎⁎	Annual crops	Agricultural lands	Vertic Calcixerertps
SYSW33	36.4669	32.6992	782	270	1	Basalt⁎⁎	Annual crops	Agricultural lands	Chromic Haploxererts
SYSW34	36.422	32.6638	772	250–200	1	Basalt⁎⁎	Annual crops	Agricultural lands	Chromic Calcixererts
SYSW35	36.3578	32.7539	680	250–200	1	Basalt⁎⁎	Olive trees + Annual crops	Agricultural lands	Chromic Haploxererts
SYSW36	36.3536	32.7222	680	250–200	0.5	Basalt⁎⁎	Annual crops	Agricultural lands	Chromic Haploxererts
SYSW37	36.3519	32.6383	696	250–200	2	Basalt⁎⁎	Annual crops	Agricultural lands	Typic Haploxerepts
SYSW38	36.3605	32.6899	671	250–200	0.5–2	Basalt⁎⁎	Annual crops	Agricultural lands	Chromic Calcixererts
SYS1	36.7161	32.645	1573	470–425	2	Basalt	Annual plants	Rangelands	Haploxerepts
SYS2	36.7037	32.65	1525	470–425	2–5	Basalt	Annual plants	Rangelands	Haploxerepts
SYS3	36.6917	32.6445	1490	470–425	2	Basalt	Annual plants	Rangelands	Haploxerepts
SYS4	36.6529	32.6489	1460	470–425	5–7	Basalt	Annual plants	Rangelands	Haploxerepts
SYS5	36.6719	32.6169	1455	400–350	2	Basalt	Annual plants	Rangelands	Haploxerols
SYS6	36.7152	32.6068	1440	400–350	5–7	Basalt	Annual plants	Rangelands	Haploxerols
SYS7	36.6204	32.6241	1400	400–350	5–10	Basalt	Annual plants	Rangelands	Haploxerepts
SYS8	36.5919	32.6408	1350	400–350	5	Basalt	Annual plants	Rangelands	Haploxerols
SYS9	36.5722	32.6304	1320	400–350	5–7	Basalt	Annual plants	Rangelands	Xerorthents
SYS10	36.7117	32.5796	1350	300–250	2	Basalt	Annual plants	Rangelands	Xerorthents
SYS11	36.6417	32.5915	1250	300–250	0.5–2	Basalt	Annual plants	Rangelands	Haploxererts
SYS12	36.6005	32.5859	1200	300–250	5	Basalt	Annual plants	Rangelands	Haploxerols
SYS13	36.6086	32.5524	1200	300–250	1	Basalt	Annual plants	Rangelands	Haploxerols
SYS14	36.7364	32.5067	1150	300–250	2–5	Basalt	Annual plants	Rangelands	Haploxerepts
SYS15	36.7089	32.5053	1150	250–200	1	Basalt	Annual plants	Rangelands	Haploxerols
SYS16	36.666	32.5189	1200	250–200	1	Basalt	Annual plants	Rangelands	Xerorthents
SYS17	36.6079	32.495	1050	250–200	2–5	Basalt	Annual plants	Rangelands	Haploxererts
SYD1	3,600,005	32.9116	559	500	0.5	Basalt	Annual crops (wheat)	Agricultural lands	Typic Haploxererts
SYD2	36.0111	32.8884	557	450–400	1–2	Basalt⁎⁎	Annual crops (wheat)	Agricultural lands	Vertic Haploxererts
SYD3	36.028	32.8583	544	450–400	1–2	Basalt⁎⁎	Vineyards+ Annual crops	Agricultural lands	Typic Haploxererts
SYD4	36.0606	32.8522	535	450–400	2–5	Basalt⁎⁎	Vegetables	Agricultural lands	Typic Haploxererts
SYD5	36.0867	32.8683	587	500–350	0.5	Basalt⁎⁎	Annual crops (wheat)φ	Agricultural lands	Typic Haploxererts
SYD6	36.08	32.9	590	400–350	2–5	Basalt⁎⁎	Annual crops (Barely) φ	Agricultural lands	Typic Haploxererts
SYD7	36.0622	32.8208	455	400–350	2–5	Basalt⁎⁎	Annual crops	Agricultural lands	Typic Haploxerepts
SYD8	36.0889	32.8306	525	350	5	Basalt⁎⁎	Annual crops (wheat) φ	Agricultural lands	Vertic Haploxerepts
SYD9	36.1294	32.8306	516	350	5–10	Basalt⁎⁎	Annual crops (wheat) φ	Agricultural lands	Typic Haploxerepts
SYD10	36.1169	32.8583	559	350	5	Basalt⁎⁎	Annual crops	Agricultural lands	Typic Haploxerepts
SYD11	36.1769	32.8706	525	300	0.5	Basalt⁎⁎	Vineyard (7 years)	Agricultural lands	Typic Haploxerepts
SYD12	36.1731	32.8336	536	350–300	Flat	Basalt⁎⁎	Annual crops (wheat) φ	Agricultural lands	Typic Haploxererts
SYD13	36.2297	32.8633	570	350–300	Flat	Basalt⁎⁎	Vegetables	Agricultural lands	Vertic Haploxerepts
SYD14	36.0383	32.8053	494	300	0.5	Basalt⁎⁎	Annual crops (wheat) φ	Agricultural lands	Typic Haploxerepts
SYD15	36.0153	32.7792	479	300	0.5	Basalt⁎⁎	Annual crops (wheat) φ	Agricultural lands	Typic Haploxerepts
SYD16	36.0803	32.7792	491	300	0.5	Basalt⁎⁎	Annual crops (wheat) φ	Agricultural lands	Chromic Calcixererts
SYD17	36.1289	32.7856	510	300	2–5	Basalt⁎⁎	Vineyard (7 years)	Agricultural lands	Chromic Calcixererts
SYD18	36.1736	32.7681	445	300	2–5	Basalt⁎⁎	Vineyard (7 years)	Agricultural lands	Chromic Haploxererts
SYD19	36.2108	32.7733	581	250–200	2	Basalt⁎⁎	Annual crops	Agricultural lands	Typic Calcixerepts
SYD20	36.1667	32.7483	560	250–200	2–5	Basalt⁎⁎	Annual crops (wheat) φ	Agricultural lands	Chromic Haploxererts
SYD21	36.0914	32.7472	489	250–200	2–5	Basalt⁎⁎	Annual crops (wheat) φ	Agricultural lands	Chromic Haploxererts
SYD22	36.0456	32.7542	472	250–200	2–5	Basalt⁎⁎	Annual crops (wheat) φ	Agricultural lands	Chromic Calcixererts
SYD23	36.0219	32.7239	472	300–250	5	Basalt⁎⁎	Annual crops (wheat) φ	Agricultural lands	Chromic Haploxererts
SYD24	36.0494	32.6936	458	280	Flat	Basalt⁎⁎	Annual crops (wheat)	Agricultural lands	Typic Haploxerepts
SYD25	36.0228	32.6622	460	300–250	5	Basalt⁎⁎	Annual crops (wheat) φ	Agricultural lands	Chromic Calcixererts
SYD26	36.1092	32.7153	540	300–250	2–5	Basalt⁎⁎	Annual crops (wheat) φ	Agricultural lands	Chromic Calcixererts
SYD27	36.1719	32.7447	548	275–250	2–5	Basalt⁎⁎	Annual crops (wheat) φ	Agricultural lands	Typic Haploxerepts
SYD28	36.2203	32.7111	571	250	Flat	Basalt⁎⁎	Annual crops (Barely) φ	Agricultural lands	Vertic Haploxerepts
SYD29	36.1708	32.6775	592	270	Flat	Basalt⁎⁎	Annual crops (Barely) φ	Agricultural lands	Typic Haplocambids
SYD30	36.1317	32.6575	514	255	2	Basalt⁎⁎	Annual plants	Rangelands	Typic Haplocambids
SYD31	36.0906	3.3E+08	506	270	5	Basalt⁎⁎	Annual crops	Agricultural lands	Typic Haploxererts
SYD32	36.1697	32.6425	584	250	Flat	Basalt⁎⁎	Annual crops (Barely) φ	Agricultural lands	Typic Haplocalcids
SYD33	36.2242	32.6481	593	250	1	Basalt⁎⁎	Annual crops (Barely) φ	Agricultural lands	Xeric Haplocalcids
SYD34	36.1808	32.6325	576	250	2	Basalt⁎⁎	Annual plants	Rangelands	Chromic Calcixererts
SYD35	36.1775	32.6053	537	250–150	1	Basalt⁎⁎	Annual plants	Rangelands	Typic Haplocalcids
SYD36	36.0542	32.6219	549	220	0–0.5	Basalt⁎⁎	Annual crops (Barely) φ	Agricultural lands	Typic Haplocalcids
SYD37	36.0969	32.5942	548	200–150	3	Calcareous rock	Annual plants	Rangelands	Lithic Petrocalcids
SYD38	36.125	32.5917	531	200	2	Basalt⁎⁎	Annual plants	Rangelands	Vertic Haplocalcids
SYD39	36.1324	32.6247	532	200–150	2	Basalt⁎⁎	Annual plants	Rangelands	Vertic Haplocalcids
SYD40	36.0983	32.5725	554	200	3	Calcareous & Basalt	Olive trees + Annual crops	Agricultural lands	Typic Haplocalcids
SYD41	36.111	32.5625	571	250–150	2	Basalt⁎⁎	Annual crops	Agricultural lands	Lithic Petrocalcids
SYD42	36.0972	32.5444	592	250–150	3	Calcareous & Basalt	Annual plants	Rangelands	Typic Haplocalcids
SYD43	36.1386	32.5547	597	200–150	4	Calcareous rock	Olive trees	Agricultural lands	Typic Haplocalcids
SYD44	36.155	32.575	581	200–150	2	Calcareous & Basalt	Annual plants	Rangelands	Typic Haplocalcids
SYD45	36.1614	32.5414	618	200–150	2	Calcareous rock	Olive trees	Agricultural lands	Typic Haplocalcids
SYD46	36.1744	32.5344	586	200–150	3	Calcareous & Basalt	Annual plants	Rangelands	Typic Haplocalcids
SYD47	36.1969	32.5522	575	200–150	3	Calcareous & Basalt	Annual crops (Barely) φ	Agricultural lands	Typic Haplocalcids

⁎ According to *Soil Taxonomy* (Soil Survey Staff, 2014).

⁎⁎ Transported φ: Rain-fed agriculture.

**Table 2:** Morphological characteristics for the studied representative soil profiles from the study area.

Profile	Depth (cm)	Matrix color	Texture Field	Structure	Roots	HCl reaction	Biological activity	Horizon boundary	Diagnostic horizon(s)
SYDJ1	0–20	7.5YR 3/4	C	1,F,GR	1	0	H	C	Mollic
	20–55	7.5YR 3/4	C	2,M,SBK,	1	0	H	C	
	55–89	Rock							
SYDJ2	0–30	7.5YR 3/2	CL	1,F,GR	1	0	H	C	Mollic
	30>	Rock							
SYDJ3	0–10	7.5YR 3/4	C	1,F,GR	1	0	H	R	Mollic
	10–37	7.5YR 3/4	C	2,M,SBK,	2	0	H	R	
	50–90	7.5YR 3/4	C	2,M,SBK,	2	0	H	R	
	90	Rock							
SYDJ4	0–15	7.5YR 3/4	C	2,F,GR	1	0	H	R	Mollic
	15–55	7.5YR 3/4	C	3,M,SBK,	1	0	H	R	
SYDJ5	0–20	7.5YR 4/6	C	1,M,GR	2	0	H	A	Ochric
	20–45	7.5YR 4/6	C	1,F,GR	2	0	H	A	
	45	Rock							
SYSW1	18 –0	7.5YR 3/2	C	1,F,GR	3	0	M	R	
	18–40	7.5YR 3/3	C	2,M,ABK,	2	0	M	R	
	40–100	7.5YR 3/2	C	3,S,ABK,	1	0	0		
SYSW2	0–22	7.5YR 3/4	C	2,F,GR	3	0	M	R	
	22–45	7.5YR 3/2	C	2,M,ABK,	3	0	M	R	
	45–75	7.5YR 4/4	C	3,M,ABK,	3	0	M		
SYSW3	0–25	7.5YR 3/4	C	2,F,GR	3	0	M	W	
	25–90	7.5YR 3/4	C	1,M,ABK,	1	0	M	W	
SYSW4	0–22	10 YR4/3	C	1,M,GR	2	0	M	R	
	22–50	10 YR4/3	C	2,M,ABK,	1	0	M	R	
	50–85	Rock+Soil							
SYSW5	0–12	7.5YR 3/2	C	2,F,GR	2	1	M	R	
	12–50	7.5YR 3/2	C	2,F,P	1	0	M		
SYSW6	0–18	7.5YR 3/2	C	2,F,GR	3	0	WE	R	
	18–60	7.5YR 3/2	C	1,M,GR	3	0	M	R	
	60–90	7.5YR 3/2	C	2,M,ABK,	3	0	WE		
SYSW7	0–17	7.5YR 3/4	C	2,M,GR	1	0	M	R	
	17–30	7.5YR 3/4	C	2,M,MA	1	0	M	W	
	30–50	7.5YR 3/2	C	3,M,MA	1	0	M	W	
	50–110	Rock+Soil							
SYSW8	0–12	7.5YR 3/4	C	2,M,GR	2	0	M	R	
	12–50	7.5YR 3/4	C	NA	1	0	NA	A	
SYSW9	0–22	7.5YR 3/4	C	2,M,GR	3	0	2	R	
	22–35	7.5YR 3/4	C	2,M,ABK,	2	0	1	R	
	35–60	Rock+Soil							
SYSW10	0–25	10YR 4/3	C	2,M,GR	3	0	M	R	
	25–40	10 YR 3/3	C	2,M,ABK,	2	0	M	R	
	40–90	10 YR 3/3	C	2,M,P	1	0	M		
SYSW11	0–25	5 YR 3/4	C	2,M,GR	3	1	M	A	
	25–80	5 YR 4/4	C	2,F,ABK,	2	1	M	R	
	80–100	5 YR 3/6	C	2,M,ABK,	2	1	M		
SYSW12	0–18	7.5YR 3/2	C	2,M,GR	2	NA	M	R	
	18–55	7.5YR 3/2	C	2,F,ABK,	2	1	M	W	
	55–120	7.5YR 3/2	C	2,M,ABK,	1	1	M	W	
SYSW13	0–20	7.5YR 3/4	C	2,S,GR	0	0	M	R	
	20–45	7.5Y4/4	C	2,S,ABK,	1	1	M	W	
	45–70	7.5YR 3/4	C	2,S,ABK,	0	1	WE	W	
SYSW14	0–18	7.5Y4/4	C	2,F,GR	2	0	M	R	
	18–40	7.5Y4/4	C	3,S,ABK,	2	1	M	W	
	40–65	7.5Y3/4	C	NA	2	1	M	W	
	65–100	7.5Y3/4	C	NA	1	1	NA		
SYSW15	0–18	5YR 3/2	C	2,F,GR	1	0	G	R	
	18–50	5YR 3/2	C	3,M,ABK,	1	0	M	R	
	50–90	5YR 3/2	C	3,S,ABK,	0	0	WE		
SYSW16	0–18	7.5 YR 4/6	C	2,F,GR	2	1	M	R	
	18–35	7.5 YR 3/4	C	NA	3	1	M	R	
	35–70	7.5 YR 3/4	C	2,F,ABK,	3	0	M	R	
	100–70	7.5 YR 3/4	C	3,M,ABK,	3	0	M		
SYSW17	25–0	7.5YR3/4	C	3,M, GR	3	1	M	G,W	
	50–25	7.5YR3/4	C	3,M, B	2	1	WE	G,W	
	80–50	7.5YR3/4	C	3,M, B	1	1	WE		
SYSW18	15–0	7.5YR3/4	C	2,F, GR	1	1	WE	G,SM	
	40–15	7.5YR3/4	C	3,M, B	1	1	WE	G,SM	
	85–40	7.5YR3/2	C	3,M, B	1	1	WE	G,SM	
	110–85	7.5YR3/2	C	3,M, B	1	1	WE		
SYSW19	20–0	5YR3/2	C	3,M, GR	2	1	M	G,W	
	50–20	5YR3/2	C	3,M, B	2	1	M	C,W	
	100–50		C	3,M, B	1	1	WE		
SYSW20	18–0	7.5YR3/4	C	2,F, GR	1	1	WE	G,SM	
	50–18	7.5YR3/5	C	3,M, B	1	1	WE	G,SM	
	110–50	7.5YR3/2	C	3,M, B	1	1	WE		
SYSW21	14–0	7.5YR3/4	C	2,F, GR	2	1	M	G,SM	
	50–14	7.5YR3/4	C	3,M, B	1	1	M	G,SM	
	70–50	7.5YR4/4	C	3,M, B	1	1	M	G,SM	
	95–70	7.5YR4/4	C	3,M, B	1	1	M	G,SM	
	95		C	ROCK	0	0			
SYSW22	15–0	7.5YR4/4	C	2,F, GR	1	2	M	G,SM	
	60–15	7.5YR3/4	C	3,M, B	1	2	M	G,SM	
	110–60	7.5YR3/4	C	3,M, B	0	2	WE	G,SM	
		7.5YR3/4	C	3,M, B	0	2	WE		
SYSW23	22–0	7.5YR4/4	C	2,F, GR	2	2	M	C,SM	
	60–22	7.5YR4/4	C	3,M, B	1	2	WE	G,SM	
	95–60	7.5YR4/4	C	3,M, B	0	2	WE	G,SM	
			C	ROCK	0	2	WE		
SYSW24	0–15	7.5YR4/4	C	2,F, GR	1	1	M	G,W	
	15–60	7.5YR4/3	C	3,M, B	1	1	WE	G,SM	
	60–110	7.5YR4/3	C	3,M, B	0	2	WE	G,SM	
SYSW25	0–20	5YR3/4	C	1,F, GR	1	1	M	G,SM	
	20–50	5YR3/2	C	2,M,SBK	0	1	WE	G,SM	
	50–100	5YR3/4	C	2,M,SBK	0	1	WE	G,SM	
SYSW26	16–0	7.5YR4/4	C	3,F, GR	1	1	M	G,SM	
	40–16	7.5YR3/4	C	3,M,SBK	1	1	WE	G,SM	
	80–40	7.5YR3/4	C	3,M,SBK	0	1	WE	G,SM	
	100–80	7.5YR3/4	C	3,M,SBK	0	1	WE	C.W	
			C	ROCK	0	2	WE		
SYSW27	0–10	5YR3/4	C	2,F, GR	1	1	M	G,SM	
	10–50	5YR3/2	C	2,M, B	0	1	WE	C.W	
	90–50	5YR3/2	C	2,M, B	0	1	WE		
SYSW28	18–0	2.5YR3/6	C	1,F, GR	1	1	M	G,SM	
	90–18	2.5YR3/6	C	3,M,SBK	1	1	WE	G,SM	
	120–90	2.5YR3/6	C	3,M,SBK	1	2	WE	G,SM	
SYSW29	15–0	7.5YR3/4	C	2,F, GR	3	1	M	G,SM	
	50–15	7.5YR3/4	C	2,M, B	2	1	M	C.W	
	130–50	7.5YR3/4	C	ROCK	0	2			
SYSW30	20–0	7.5YR3/4	C	2,F, GR	1	1	M	G,SM	
	55–20	7.5YR3/4	C	2,M, B	1	1	WE	G,SM	
	100–55	7.5YR3/4	C	2,M, B	0	1	WE		
SYSW31	15–0	7.5YR3/4	C	2,F, GR	1	1	M	G,SM	
	60–15	7.5YR3/4	C	2,M, B	1	1	WE	G,SM	
	110 - 60	7.5YR3/4	C	2,M, B	0	1			
SYSW32	0–10	7.5YR4/4	C	2,F, GR	1	1	M	C,SM	
	44,134	7.5YR4/6(w)	C	2,M, B	0	1	WE	G,SM	
	60–30	7.5YR4/6(w)	C	2,M, B	0	1	WE	G,SM	
	120–60	7.5YR3/4(w)	C	2,M, B	0	1	WE	G,SM	
SYSW33	18–0	7.5YR4/4	C	1,F, GR	2	2	M	G,W	
	50–18	7.5YR4/4	C	2,M, B	1	2	M	G,SM	
	90–50	7.5YR3/4	C	2,M, B	1	2	M	G,SM	
	100–90	7.5YR3/4	C	ROCK	1	3	M	C,SM	
	100		C		0	3			
SYSW34	18–0	7.5YR4/4	C	2,M, GR	1	2	M	G,SM	
	50–18	7.5YR4/4	C	3,M,SBK	1	2	M	G,SM	
	80–50	7.5YR4/4(W)	C	3,M,B	0	3	WE	G,SM	
	150–80	7.5YR4/6(W)	C	3,M,B	0	3	WE	C,SM	
	150–200		C	ROCK	0	3			
SYSW35	25–0	7.5YR4/6(W)	C	2,M, GR	2	2	M	C,SM	
	50–25	7.5YR4/6(W)	C	3,M,SBK	2	2	M	G,SM	
	90–50	7.5YR4/4(W)	C	3,M,SBK	1	2	WE	G,SM	
	180–90	7.5YR4/4(W)	C	3,M,SBK	0	3	WE	G,SM	
	220–180	7.5YR4/4(W)	C	3,M,SBK	0	3	WE		
SYSW36	15–0	7.5YR4/4	C	2,F, GR	0	0	G	C, SM	
	50–15	7.5YR3/4	C	3,M,SBK	3	0	M	C, SM	
	100–50	7.5YR3/4	C	3,S,SBK	2	0	M		
SYSW37	20–0	7.5YR4/4	C	2,F, GR	2	0	M	C, SM	
	60–20	7.5YR4/4	C	3,S,SBK	3	0	M	C, SM	
	120–60	Rock+Soil							
SYSW38	18–0	5 YR 4/6	C	1,F, GR	2	2	M	C, SM	
	50–18	5 YR 4/6	C	2,F,SBK	2	2	M	C, W	
	90–50	5 YR 4/6	C	2,M,SBK	1	3	NA	IR	
	130–90	5 YR 3/4	C	2,M,SBK	1	3	NA	C, W	
	200–130	5 YR 3/4	C	3,M,SBK	0	3	NA	C, W	
	260–200	5 YR 3/4	C	3,M,SBK	0	3	NA	–	
SYS1	0–30	5 YR 3/4	cl	1,F, GR	3	1	M	G, W	Ochric
	30–60	5 YR 3/3	C	2,M, B	3	1	WE	G, W	
	60–80	5 YR 3/3	C	3,M, B	0	1	WE		
SYS2	0–20	7.5 YR 3/4		2,F, GR	3	1	M	G, W	Ochric
	20–40	7.5 YR 3/2		2,M, B	2	1	WE	G, W	
	40–80	7.5 YR 3/2		2,M, B	1	1	WE		
SYS3	0–25	10YR3/3	C	1,F, GR	2	1	M	G, W	Ochric
	25–50	10YR3/3	C	2,M, B	1	1	M	G, W	
	50–90	10YR3/3	C	2,M, B	1	1	WE		
SYS4	0–30	2.5YR4/8	C	2,F, GR	2	1	M	G,SM	Ochric
	30–70	2.5YR4/6	C	2,M, B	2	1	M	G,SM	
	70–90	2.5YR3/6	C	2,M, B	0	1	WE	–	
SYS5	0–20	7.5 YR 3/4(W)	CL	3,F, GR	3	1	M	D,IR	Mollic
	20–50	7.5 YR 3/4(W)	C	2,F,B	2	1	M	G, W	
	50–90	7.5 YR 3/4(W)	C	3,M, B	1	1	WE		
SYS6	0–20	7.5 YR 4/4	C	2,F, GR	2	1	M	G, W	Mollic
	20–40	7.5 YR 4/4	C	2,M, B	1	1	WE	G, SM	
	40–100	7.5 YR 3/4	C	2,M, B	0	1	WE		
SYS7	0–30	7.5 YR 4/6	CL	1,F, GR	2	1	M	G,SM	Ochric
	30–70	7.5 YR 4/4	CL	1,F,B	1	1	WE	G,SM	
	70–110	7.5 YR 4/4	CL	1,M, B	0	1	WE		
SYS8	0–20	7.5 YR 4/4	C	2,F, GR	2	1	M	G, W	Mollic
	20–50	7.5 YR 3/4	C	2,F, B	2	1	WE	G, W	
	50–110	7.5 YR 3/4	C	2,M, B	0	1	WE		
SYS9	0–20	10YR3/3	L	2,F, GR	2	1	M	G, W	Ochric
	20–50	10YR3/3	SCL	1,F, B	1	1	WE	G, W	
	50–90	10YR3/3	SCL	1,M,B	0	1	WE		
SYS10	0–25	7.5 YR 3/4	CL	2,F, GR	2	1	M	G, W	Ochric
	25–50	7.5 YR 3/2	CL	1,M,B	2	1	M	G, W	
	50–100	7.5 YR 3/3	CL	1,M,B	0	1	WE		
SYS11	0–25	7.5 YR 3/4	C	2,F, GR	1	2	M	G, W	Ochric
	25–60	7.5 YR 3/2	C	3,M,B	1	2	WE	G, W	
	60–100	7.5 YR 3/3	C	3,M,B	0	2	WE		
SYS12	0–20	7.5 YR 4/4	LS	1,F, GR	2	2	M	G,W	Mollic
	20–50	7.5 YR 3/4	LS	1,F,B	1	2	M	G,W	
	50–100	7.5 YR 3/4	LS	1,F,B	0	2	WE		
SYS13	0–30	2.5 YR 3/6		2,F, GR	3	1	M	G,W	Mollic
	30–60	2.5 YR 3/4		2,M, B	2	2	M	G,W	
	60–100	2.5 YR 3/4		2,M, B	1	2	WE		
SYS14	0–20	5 YR 3/4		1,F, GR	2	2	M	G,W	Ochric
	20–40	5 YR 3/4		1,F,B	1	1	WE	G,W	
	40–110	5 YR 3/4		2,M, B	1	1			
SYS15	0–20	7.5 YR 4/2		2,F, GR	2	1	M	G,W	Mollic
	20–40	7.5 YR 3/2		2,M, B	1	1	WE	G,W	
	40–90	7.5 YR 3/2			0	1	WE		
SYS16	0–20	7.5 YR 3/4		2,F, GR	2	2	M	G,W	Ochric
	20–40	7.5 YR 3/5		2,F,B	1	2	WE	G,W	
	40–100	7.5 YR 3/4		2,F,B	0	1	–	–	
SYS17	0–20	7.5 YR 3/4	C	3,F, GR	2	1	M	G,SM	
	20–50	7.5 YR 3/4	C	3,M, B	2	1	WE	G,SM	
	50–120	7.5 YR 3/4	C	3,M, B	1	1	WE	–	
								
SYD1	20–0	5 YR 3/4		3,M, GR	1	1	WE	G,W	
	60–20	5 YR 3/4		3,M, B	1	1	WE	G,W	
	100–60	5 YR 3/4		3,M, B	0	1	WE		
SYD2	20–0	5 YR 4/4		3,M, GR	1	1	M	G,W	
	60–20	5 YR 4/4		3,M, aBk	1	1	WE	G,SM	
	100–60	5 YR 4/4		3,M, aBk	0	1	WE		
SYD3	15–0	5 YR 4/4		3,M, GR	1	1	WE	G,SM	
	60–15	5 YR 4/4		3,M, B	1	1	WE	G,SM	
	110–60	5 YR 4/4		3,M, B	0	1	WE		
SYD4	18–0	5 YR 4/4		3,M, GR	1	1	M	G,SM	
	60–18	5 YR 4/4		3,M, B	1	1	WE	G,SM	
	110–60	5 YR 4/4		3,M, B	0	1	WE		
SYD5	20–0	5 YR 4/4		3,M, GR	3	1	M	G,W	
	60–20	5 YR 4/4		3,M, aBk	1	1	WE	G,W	
SYD6	20–0	5 YR 4/4		3,M, GR	3	1	M	G,W	
	55–20	5 YR 4/4		3,M, aBk	1	1	WE	G,W	
	80–55	5 YR 4/4		3,M, aBk	0	1	WE		
SYD7	0–12	5 YR 5/4		3,M, GR	2	2	M	G,SM	
	40–12	5 YR 5/4		Platy	0	2	WE	G,SM	
	85–40	5 YR 5/4		Platy	0	2	WE	G,SM	
	130–85	5 YR 5/4		Platy	0	2	WE		
SYD8	0–10	5 YR 5/4		3,M, GR	2	1	M	G,SM	
	38–10	5 YR 5/4		Platy	0	1	WE	G,SM	
	80–38	5 YR 5/4		Platy	0	2	WE	G,SM	
	125–80	5 YR 5/4		Platy	0	2	WE		
SYD9	30–0	5 YR4/4		3,M, GR	1	1	M	G,SM	
	60–30	5 YR4/4		3,M, B	0	2	WE	G,SM	
	100–60	5 YR4/4		3,M, B	0	2	WE	G,SM	
	150–100	5 YR4/4		3,M, B	0	2	WE		
SYD10	22–0	5 YR4/4		3,M, GR	1	1	M	G,SM	
	55–22	5 YR4/4		3,M, B	1	1	WE	G,SM	
	100–55	5 YR4/4		3,M, B	0	1	WE	G,SM	
	140–100	5 YR4/4		3,M, B	0	1	WE		
SYD11	25–0	7.5 YR 4/4		3,M, GR	1	1	M	G,SM	
	50–25	7.5 YR 4/4		3,M, B	0	1	WE	G,SM	
	100–50	7.5 YR 4/4		3,M, B	0	1	WE	G,SM	
	130–100	7.5 YR 4/4		3,M, B	0	1	WE		
SYD12	20–0	7.5 YR 5/4		3,M, GR	1	1	M	G,SM	
	85–20	7.5 YR 5/4		3,M, B	0	1	WE	G,SM	
	110–85	7.5 YR 5/4		3,M, B	0	1	WE		
SYD13	0–10	7.5 YR 4/4		3,M, GR	1	1	M	G,SM	
	55–10	7.5 YR 4/4		3,M, B	0	1	WE	G,SM	
	55–120	7.5 YR 4/4		3,M, B	0	1	WE		
SYD14	25–0	5 YR 5/6		3,M, GR	2	1	M	G,SM	
	70–25	5 YR 5/6		3,M, B	1	1	WE	G,SM	
	120–70	5 YR 5/6		3,M, B	0	1	WE		
SYD15	0–10	5 YR 5/6		3,M, GR	1	1	M	G,SM	
	50–10	5 YR 5/6		3,M, B	0	1	WE	G,SM	
	100–50	5 YR 5/6		3,M, B	0	1	WE		
SYD16	10–0	5 YR 4/6		3,M, GR	1	1	M	G,SM	
	45–10	5 YR 4/6		3,M, aBk	0	1	WE	G,SM	
	70–45	5 YR 4/6		3,M, B	0	1	WE		
	120–70								
SYD17	0–12	5 YR 4/6		3,M, GR	1	1	M	G,SM	
	50–12	5 YR 4/6		3,M, B	0	1	WE	G,SM	
	80–50	5 YR 4/6		3,M, B	0	1	WE		
	135–80								
SYD18	0–10	5 YR 4/6		3,M, GR	1	1	M	G,SM	
	40–10	5 YR 4/6		3,M, aBk	0	1	WE	G,SM	
	110–40	5 YR 4/6		3,M, B	0	2	WE		
SYD19	30–0	10 YR 5/6		3,M, aBk	1	1	WE	G,SM	
	90–30	10 YR 5/6		3,M, aBk	0	2	WE	G,SM	
	140–90	10 YR 5/6		3,M, aBk	0	2	WE		
SYD20	0–10	5 YR 4/6		3,M, GR	1	1	M	G,SM	
	50–10	5 YR 4/6		3,M, aBk	0	2	WE	G,SM	
	110–50	5 YR 4/6		3,M, aBk	0	2	WE		
SYD21	0–12	5 YR 5/6		3,M, GR	2	1	M	G,SM	
	50–12	5 YR 5/6		3,M, aBk	0	2	WE	G,SM	
	120–50	5 YR 5/6		3,M, aBk	0	2	WE		
SYD22	0–12	5 YR 5/6		3,M, GR	2	1	M	G,SM	
	60- 12	5 YR 5/6		3,M, aBk	0	3	WE	G,SM	
	90 −60	5 YR 5/6		3,M, aBk	0	3	WE		
	130- 90								
SYD23	32–0	5 YR 4/6		3,M, aBk	1	1	M	G,W	
	75–32	5 YR 4/6		3,M, aBk	0	3	WE	G,W	
	120–75	5 YR 4/6		3,M, aBk	0	3	WE		
SYD24	30–0	5 YR 5/6		3,M, aBk	1	1	M	G,W	
	65–30	5 YR 5/6		3,M, aBk	0	2	WE	G,W	
	110–65	5 YR 5/6		3,M, aBk	0	2	WE		
SYD25	28–0	5 YR 5/6		3,M, aBk	1	1	M	G,W	
	60–28	5 YR 5/6		3,M, aBk	0	2	WE	G,W	
	110–60	5 YR 5/6		3,M, aBk	0	2	WE		
SYD26	10–00	5 YR 5/6		3,M, aBk	1	1	M	G,SM	
	50–10	5 YR 5/6		3,M, aBk	0	2	WE	G,SM	
	110–50	5 YR 5/6		3,M, aBk	0	2	WE		
SYD27	20–0	7.5 YR 4/6		2,F, GR	1	1	M	G,SM	
	50–20	7.5 YR 4/6		2,F,B	0	2	WE	G,SM	
	100–50	7.5 YR 4/6		2,F,B	0	2	WE		
	150–100	Rock+Soil							
SYD28	18–0	7.5 YR 4/4	CL	2,F,GR	2	1	M	C, SM	
	50–18	7.5 YR 4/4	CL	2,M, aBk	0	2	WE	C, SM	
	80–50	7.5 YR 4/4	C	2,M, aBk	0	3	NA	C, SM	
	140–80	7.5 YR 4/4	C	3,M, aBk	0	3	NA	C, SM	
SYD29	25–0	7.5 YR 4/6	CL	2,F,GR	2	2	M	C, SM	
	60–25	7.5 YR 4/6	CL	2,M, aBk	0	3	M	C, SM	
	90–60	7.5 YR 4/6	C	2,M, aBk	0	3	NA	C, SM	
	130–90	7.5 YR 4/6	C	3,M, aBk	0	3	NA	C, SM	
SYD30	22–0	5 YR 4/4	L	2,F,GR	0	1	M	C, SM	
	60–22	5 YR 4/4	C	2,M, aBk	0	3	M	C, SM	
	100–60	5 YR 4/4	C	3,M, aBk	0	3	NA	C, SM	
SYD31	33 –0	5 YR 4/4	CL	1,F,GR	1	2	M	C, SM	
	105–33	5 YR 4/4	C	2,M, aBk	0	2	M	C, SM	
	160–105	5 YR 4/4	C	2,M, aBk	0	3	WE		
SYD32	20–0	7.5 YR 4/6	CL	1,F,GR	1	3	M	G,SM	
	50–20	7.5 YR 4/6	CL	2,M, aBk	0	3	M	G,A	
	100–50	7.5 YR 4/6	CL	3,M, aBk	0	3	WE	G,SM	
	140–100	7.5 YR 4/6	CL	3,3, aBk	0	3	WE	G,SM	
SYD33	30–0	7.5 YR 4/6	CL	1,F,GR	1	3	M	G,SM	
	60–30	7.5 YR 4/6	CL	2,M, aBk	0	3	M	G,SM	
	100–60	7.5 YR 4/6	CL	3,M, aBk	0	3	WE	G,W	
	140–100	7.5 YR 4/6	CL	3,3, aBk	0	3	WE	G,SM	
SYD34	18–0	7.5 YR 4/6	C	1,F,GR	0	3	M	G,SM	
	70–18	7.5 YR 4/6	C	2,M, aBk	0	2	M	G,W	
	130–70	7.5 YR 4/6	C	3,M, aBk	0	3	WE	G,SM	
SYD35	30–0	7.5 YR 4/6	CL	3,3, aBk	0	2	M	G,SM	
	100–30	7.5 YR 4/6	CL	2,M, aBk	0	2	M	G,A	
	160–100	7.5 YR 4/6	CL	3,M, aBk	0	3	WE	G,SM	
SYD36	20–0	7.5 YR 4/6	CL	2,F,GR	0	1	M	G,SM	
	70 –20	7.5 YR 4/6	CL	2,M, aBk	0	2	M	G,A	
SYD37	20–0	7.5 YR 4/3	CL	1,F,GR	0	2	WE	G,SM	
	20+	Rock							
SYD38	30–0	5 YR 4/4	L	2,F,GR	0	2	WE	C, SM	
	80–30	5 YR 4/4	C	2,F, aBk	0	3	WE	C, A	
	120–80	7.5 YR 4/4	C	3,M, aBk	0	3	WE		
SYD39	20–0	7.5 YR 5/4	CL	1,F,GR	1	2	WE	C, SM	
	50–20	7.5 YR 5/4	CL	1,M, aBk	1	2	WE	G,A	
	110–50	7.5 YR 5/4	CL	3,M, aBk	0	3	WE		
SYD40	30–0	10 YR 6/6	SiL	1,F,GR	1	1	M	C, SM	
	60–30	10 YR 5/6	L	1,M, aBk	0	2	WE	G,W	
	100–60	10 YR 5/6	L	2,M, aBk	0	3	NA	G,W	
	140–100	10 YR 6/6	L	3,M, aBk	0	3	NA		
SYD41	0–12	7.5 YR 4/3	CL	1,F,GR	0	2	WE	G,SM	
	12 <	Rock							
SYD42	20–0	10 YR 6/6	SiCL	1,F,GR	1	3	M	C, SM	
	50–20	10 YR 5/6	CL	1,M, aBk	1	2	M	G,W	
	80–50	10 YR 5/6	CL	2,M, aBk	1	3	NA	G,W	
	120–80	10 YR 6/6	CL	3,M, aBk	0	3	NA		
SYD43	25–0	10 YR 6/6	SiL	1,F,GR	1	3	M	C, SM	
	25 <	Rock							
SYD44	15–0	5 YR 5/6	CL	1,F,GR	1	3	M	C, SM	
	40–15	5 YR 5/6	CL	2,M,SBK,	1	3	M	G,W	
	80–40	5 YR 5/6	CL	2,M,SBK,	1	3	M	G,W	
	120–80	5 YR 5/6	CL	3,M,SBK,	0	3	M		
SYD45	25–0	10 YR 6/6	SiL	1,F,GR	1	3	M	C, SM	
	< 25	Rock							
SYD46	20–0	10 YR 6/6	SiL	1,F,GR	1	3	M	C, SM	
	75–20	10 YR 5/6	SiL	2,M,SBK,	1	2	M	G,W	
	115–75	10 YR 6/6	SiL	2,M,SBK,	1	3	WE	G,W	
	140–115	10 YR 6/6	SiL	3,M,SBK,	0	3	NA		
SYD47	25–0	10 YR 6/6	L	1,F,GR	1	2	M	C, SM	
	65–25	10 YR 6/6	L	2,M,ABK,	0	2	WE	G,W	
	100–65	10 YR 6/6	L	3,M,SBK,	0	2	WE		

a Field texture: SL = sandy loam; CL = clay loam.

b Structure: 1 = weak; 2 = moderate; 3 = strong; *F* = fine; *M*= medium; *C* = coarse; P: prismatic; SBK = subangular blocky; *B*= blocky, ABK = angular blocky; GR = granular; SG = Single grain; MA = massive.

c Roots abundance: 0 = none; 1 = few (2–20%); 2 = common (20–50%); 3 = many (>50%).

d HCl effervescence: 1 = slight; 2 = moderate; 3 = strong; 4 = very strong.

e Biological activity: *N* = none; *W* = weak; *M* = moderate.

f Horizon boundary: *C* = clear; *D* = diffuse; *A* = abrupt; SM = smooth; IR = irregular, W: wavy.

**Table 3:** Physiochemical soil characteristics of the selected representative soil profiles in the study area.

Profile	Horizon	BD	PD	Clay	Silt	Sand	pH	EC	TOM	CaCO_3_	CEC	Ca^+2^	Mg^+2^	Na^+^	*K*^+^
		g cm^−3^		%			H_2_O	dS *m* ^−^ ^1^	%		Cmolc kg^⁻1^	mg/kg			
SYDJ1	0- 20	1.40	2.10	39.80	26.90	33.30	6.56	0.10	1.30	0.00	31.40	23.80	6.80	1.00	0.10
	20–55	1.45	2.51	39.10	33.60	27.30	6.41	0.09	1.27	0.00	30.50	22.80	6.20	1.20	0.06
	55–89	1.55	2.41	40.40	22.00	37.60	6.67	0.09	1.09	0.00	34.80	27.30	8.20	1.80	0.08
SYDJ2	0–30	1.60	2.31	31.91	49.13	18.95	6.97	0.18	1.51	0.00	30.00	22.60	8.60	1.00	0.16
SYDJ3	0–10	1.25	2.46	28.40	47.70	23.90	6.02	0.07	1.65	0.00	20.20	19.20	3.20	1.00	0.20
	10–50	1.67	2.40	49.10	26.25	24.60	6.44	0.10	1.61	0.00	32.20	24.20	6.40	1.00	0.08
	50–90	1.60	2.54	51.00	29.13	19.78	6.66	0.07	0.96	0.00	36.50	25.60	6.10	1.20	0.06
	90	1.50	2.40	46.80	33.62	19.55	6.65	0.10	0.90	0.00	31.00	28.40	6.40	1.50	0.08
SYDJ4	0–15	1.33	2.45	37.17	52.43	10.40	6.51	0.05	1.88	0.00	25.80	19.20	6.40	1.20	0.24
	15–55	1.60	2.50	37.25	49.60	12.88	6.71	0.07	1.39	0.00	27.00	20.60	8.40	1.20	0.23
SYDJ5	0–20	1.60	2.25	27.87	39.73	32.40	6.25	0.10	2.23	0.00	23.00	19.40	6.40	1.30	0.18
SYSW1	18–0	1.24	2.64	41.51	35.21	23.28	7.03	0.07	1.30	1.00	37.90	22.81	11.35	0.56	0.67
	40–18	1.39	2.58	45.51	33.21	21.28	7.11	0.03	0.87	1.00	40.90	23.40	13.99	0.60	0.37
	100–40	-	-	51.51	27.21	21.28	7.29	0.06	0.33	1.50	44.70	24.79	15.44	0.65	0.23
SYSW2	22–0	1.26	2.55	35.51	41.28	23.21	7.00	0.08	1.45	1.00	35.00	20.87	9.12	0.56	0.99
	45–22	1.40	2.59	45.51	31.28	23.21	7.09	0.04	0.87	1.00	42.10	25.37	11.67	0.53	0.68
	75–45	-	-	49.51	29.28	21.21	7.18	0.04	0.45	1.00	44.50	28.12	13.18	0.53	0.27
SYSW3	25–0	1.25	2.56	45.51	39.28	15.21	7.01	0.07	1.12	1.00	36.30	22.31	9.47	0.50	0.58
	90–25	1.33	2.57	55.51	25.28	19.21	7.12	0.10	0.67	1.00	38.30	21.92	12.86	0.55	0.27
SYSW4	22–0	1.24	2.60	35.58	27.21	37.21	7.30	0.11	1.00	1.00	35.30	22.03	9.36	0.50	1.10
	50–22	1.37	2.61	39.58	31.21	29.21	7.33	0.15	0.56	1.00	39.80	26.45	9.23	0.60	0.68
	85–50	-	-	59.58	23.21	17.21	7.57	0.13	0.22	2.00	41.70	28.85	9.42	0.58	0.54
SYSW5	12–0	1.19	2.67	31.58	39.21	29.21	6.99	0.05	0.89	1.00	33.30	19.54	9.37	0.71	0.94
	50–12	1.29	2.54	31.58	27.21	41.21	7.09	0.04	0.56	1.00	36.40	20.60	11.79	0.65	0.74
SYSW6	18–0	1.36	2.63	33.58	39.21	27.21	7.18	0.05	1.00	1.00	34.11	18.87	10.68	0.55	0.61
	60–18	1.55	2.60	41.58	33.21	25.21	7.25	0.05	0.78	1.00	36.30	20.06	12.18	0.57	0.39
	90–60	-	-	51.58	23.21	25.21	7.39	0.06	0.33	1.00	38.06	21.14	12.48	0.59	0.36
SYSW7	17–0	1.33	2.66	39.51	37.21	23.28	7.01	0.06	1.23	1.00	28.30	16.90	6.59	0.37	0.95
	30–17	1.46	2.68	41.51	35.21	23.28	7.06	0.03	1.05	1.00	30.90	17.96	8.37	0.45	0.83
	50–30	-	-	49.51	29.21	21.28	7.10	0.02	0.56	1.50	35.10	20.76	10.59	0.50	0.70
	110–50	-	-	57.51	21.21	21.28	7.12	0.05	0.33	1.50	39.90	22.87	12.41	0.75	0.56
SYSW8	12–0	1.30	2.67	44.51	30.21	25.28	7.00	0.05	0.89	1.00	34.30	18.54	10.37	0.55	0.94
	50–12	1.45	2.54	53.51	25.21	21.28	7.03	0.04	0.56	1.00	38.40	20.60	12.79	0.62	0.74
SYSW9	22–0	1.25	2.66	43.51	31.21	25.28	7.00	0.07	0.78	1.00	33.90	21.00	9.69	0.64	0.46
	35–22	1.57	2.54	48.51	28.21	23.28	7.25	0.09	0.56	1.50	34.80	20.35	10.59	0.66	0.40
	60–35	-	-	55.51	23.21	21.28	7.41	0.10	0.33	2.00	36.50	20.34	11.27	0.70	0.43
SYSW10	25–0	1.28	2.63	31.51	43.21	25.28	7.23	0.05	0.94	1.00	31.20	17.78	9.19	0.50	1.05
	40–25	1.44	2.60	42.51	34.21	23.28	7.40	0.06	0.69	1.00	34.30	19.83	10.12	0.53	0.81
	90–40	-	-	53.51	25.21	21.28	7.77	0.09	0.33	1.50	38.10	22.99	10.78	0.57	0.41
SYSW11	25–0	1.22	2.54	47.51	25.28	27.21	7.03	0.08	1.02	1.00	39.60	24.92	9.84	0.53	0.91
	80–25	1.32	2.56	57.51	19.28	23.21	7.09	0.10	0.80	1.50	43.70	30.07	8.46	0.73	0.83
	100–80	-	-	57.51	21.28	21.21	7.11	0.07	0.52	0.50	46.30	31.12	10.88	0.83	0.32
SYSW12	18–0	1.19	2.55	61.58	23.21	15.21	7.07	0.05	1.12	1.00	42.00	26.15	11.07	0.66	0.59
	55–18	1.37	2.55	63.58	23.21	13.21	7.65	0.05	0.56	1.00	43.60	27.14	10.91	0.69	0.30
	120–55	-	-	69.58	15.21	15.21	7.80	0.05	0.33	1.00	45.80	27.54	13.72	0.73	0.36
SYSW13	20–0	1.21	2.56	61.51	21.28	17.21	7.52	0.08	0.67	1.00	43.30	28.90	9.79	0.62	0.86
	45–20	1.26	2.59	63.51	13.28	23.21	7.64	0.08	0.45	1.00	45.00	30.55	9.63	0.66	0.57
	70–45	-	-	59.51	19.28	21.21	7.70	0.11	0.33	3.50	41.40	28.58	9.66	0.70	0.38
SYSW14	18–0	1.18	2.63	63.51	19.28	17.21	7.70	0.12	0.89	3.50	45.80	32.28	9.20	0.61	0.86
	40–18	1.24	2.54	63.51	19.28	17.21	7.99	0.12	0.56	6.50	46.20	31.37	9.42	0.62	0.70
	65–40	–	–	65.51	15.28	19.21	7.75	0.13	0.33	3.50	49.50	32.21	11.82	0.64	0.27
	100–65	–	–	63.51	17.28	19.21	7.84	0.13	0.33	2.50	48.10	32..27	11.01	0.74	0.36
SYSW15	18–0	1.20	2.61	53.51	22.28	24.21	7.77	0.12	0.67	4.30	41.00	28.37	8.44	0.65	0.86
	50–18	1.24	2.59	63.51	19.28	17.21	7.85	0.13	0.33	4.60	47.40	32.36	9.43	0.78	0.71
	90–50	–	–	66.51	12.28	21.21	7.84	0.15	0.22	5.00	48.20	33.41	10.04	0.98	0.66
SYSW16	18–0	1.15	2.54	57.66	25.20	17.14	7.68	0.12	0.89	5.30	41.10	25.25	10.61	0.72	1.69
	35–18	1.28	2.56	61.66	19.20	19.14	7.72	0.13	0.56	5.70	43.00	27.28	10.06	0.71	0.91
	70–35	-	-	63.66	17.20	19.14	7.87	0.13	0.33	6.40	46.20	28.49	12.67	0.76	0.51
	100–70	-	-	59.66	17.20	23.14	7.95	0.17	0.22	9.50	42.32	27.18	12.01	0.90	0.43
SYSW17	25–0	1.19	2.63	57.51	23.21	19.28	7.23	0.04	0.89	1.00	40.22	24.08	11.81	0.63	0.32
	50–25	1.27	2.58	63.51	15.21	21.28	7.40	0.22	0.45	1.50	47.30	27.53	14.45	0.66	0.22
	80–50	-	-	61.51	11.21	27.28	7.77	0.19	0.33	5.50	44.00	27.57	11.95	0.91	0.18
SYSW18	15–0	1.21	2.55	47.66	18.20	34.14	7.77	0.09	0.67	1.50	39.50	26.68	8.10	0.59	0.74
	40–15	1.35	2.56	67.66	19.20	13.14	7.85	0.08	0.33	1.50	48.50	32.65	10.75	0.63	0.63
	85–40	-	-	63.66	15.20	21.14	7.84	0.09	0.22	2.50	47.10	29.41	12.89	0.69	0.53
	110–85	-	-	63.66	15.20	21.14	7.80	0.10	0.11	6.50	45.90	29.48	11.79	0.74	0.33
SYSW19	20–0	1.17	2.62	61.66	19.20	19.14	7.72	0.10	1.12	1.50	44.90	29.80	9.68	0.65	1.12
	50–20	1.26	2.57	61.66	19.20	19.14	7.62	0.06	0.67	1.50	45.00	28.22	11.74	0.78	0.52
	100–50	-	-	63.66	15.20	21.14	7.64	0.07	0.33	1.50	45.80	27.76	12.92	0.81	0.38
SYSW20	18–0	1.18	2.56	63.66	17.20	19.14	7.80	0.11	0.56	6.50	46.00	30.61	10.29	0.65	1.01
	50–18	1.22	2.55	63.66	17.20	19.14	7.86	0.09	0.33	7.00	46.80	30.85	10.42	0.68	0.77
	110–50	-	-	63.66	17.20	19.14	7.90	0.10	0.11	7.50	47.45	31.22	12.80	0.85	0.47
SYSW21	14–0	1.24	2.56	62.58	21.21	16.21	7.66	0.15	1.00	6.50	48.30	31.43	10.79	0.49	0.89
	50–14	1.43	2.59	67.58	17.21	15.21	7.80	0.17	0.45	7.00	49.40	31.53	11.99	0.61	0.68
	70–50	–	–	65.58	17.21	17.21	7.90	0.16	0.33	7.00	49.20	30.19	13.99	0.98	0.46
	95–70	–	–	67.58	17.21	15.21	7.90	0.18	0.22	6.50	48.90	30.03	13.49	1.17	0.35
SYSW22	15–0	1.23	2.58	65.58	17.43	16.99	7.64	0.13	1.12	11.5	46.14	27.68	12.90	0.71	0.94
	60–15	1.37	2.56	65.58	19.43	14.99	7.75	0.18	0.67	12.5	46.60	29.45	11.33	0.94	0.64
	110–60	-	-	67.58	17.43	14.99	7.82	0.19	0.22	14.5	48.00	31.06	12.18	1.14	0.58
SYSW23	22–0	1.19	2.55	63.58	19.21	17.21	7.77	0.11	0.78	7.50	45.87	29.73	10.54	0.75	1.00
	60–22	1.31	2.56	65.58	17.21	17.21	7.88	0.11	0.33	8.00	48.77	31.73	11.36	0.96	0.75
	95–60	-	-	63.58	17.21	19.21	7.87	0.14	0.22	8.50	47.11	31.77	11.24	1.28	0.40
SYSW24	0–15	1.19	2.58	63.58	18.43	17.99	7.64	0.13	1.12	11.5	48.50	32.72	10.85	0.62	0.95
	15–60	1.22	2.56	65.58	19.43	14.99	7.75	0.18	0.67	12.5	51.00	33.79	11.45	0.94	0.65
	60–110	–	–	67.58	17.43	14.99	7.82	0.19	0.22	14.5	52.80	33.87	12.85	1.13	0.58
SYSW25	0–20	1.19	2.56	55.74	19.64	24.62	7.70	0.15	0.61	5.50	43.00	28.76	8.64	0.85	0.76
	20–50	1.22	2.55	66.24	14.64	19.12	7.80	0.17	0.56	6.00	47.50	30.21	10.72	1.03	0.74
	50–100	-	–	68.74	11.14	20.12	7.88	0.17	0.42	6.20	49.20	32.00	10.91	1.10	0.64
SYSW26	16–0	1.20	2.55	55.51	29.21	15.28	7.76	0.14	0.78	5.00	43.30	29.26	9.24	0.71	1.28
	40–16	1.29	2.57	65.51	19.21	15.28	7.82	0.11	0.45	6.00	47.30	29.02	12.03	0.75	1.05
	80–40	–	–	65.51	17.21	17.28	7.91	0.13	0.33	7.00	47.40	28.64	13.98	0.78	0.63
	100–80	–	–	61.51	19.21	19.28	8.01	0.15	0.11	12.00	45.80	29.90	11.42	0.81	0.43
SYSW27	0–10	1.20	2.58	53.51	25.21	21.28	7.80	0.15	0.47	9.20	40.80	24.77	10.74	0.72	0.74
	10–50	1.33	2.55	61.51	20.21	18.28	7.90	0.14	0.23	9.50	45.00	30.56	8.23	0.88	0.70
	90–50	–	–	63.51	17.21	19.28	8.00	0.16	0.19	9.70	46.30	31.84	7.75	1.22	0.59
SYSW28	18–0	1.18	2.61	61.73	22.21	16.06	7.70	0.10	1.00	14.00	45.10	27.00	13.42	0.57	1.03
	90–18	1.28	2.57	63.73	21.21	15.06	7.97	0.12	0.56	14.50	45.40	26.89	13.09	0.66	0.77
	120–90	-	-	63.73	21.21	15.06	8.08	0.19	0.33	15.50	46.00	26.35	13.60	1.08	0.56
SYSW29	15–0	1.15	2.56	51.73	27.21	21.06	7.57	0.09	1.12	2.50	39.90	24.17	9.87	0.60	1.49
	50–15	1.27	2.55	61.73	19.21	19.06	7.70	0.19	0.56	6.50	45.10	28.14	10.46	0.70	0.95
	130–50	-	-	63.73	17.21	19.06	7.89	0.13	0.33	7.50	48.00	29.56	11.55	0.88	0.65
SYSW30	20–0	1.23	2.59	57.73	23.21	19.06	7.66	0.14	1.12	4.50	42.20	26.80	9.53	0.51	1.19
	55–20	1.31	2.61	61.73	19.21	19.06	7.79	0.09	0.67	5.00	45.00	29.24	9.51	0.45	0.81
	100–55	-	-	63.73	19.21	17.06	7.84	0.10	0.45	7.00	47.70	30.62	11.61	0.58	0.60
SYSW31	15–0	1.19	2.60	59.73	17.21	23.06	7.61	0.11	1.23	10.50	44.40	26.16	11.88	0.75	1.20
	60–15	1.24	2.55	61.73	19.21	19.06	7.75	0.14	0.67	11.00	45.60	28.44	10.93	0.88	0.70
	110–60	-	-	61.73	21.21	17.06	7.89	0.18	0.33	15.50	45.80	28.29	11.95	1.00	0.47
SYSW32	0–10	1.21	2.61	57.66	21.20	21.14	7.95	0.12	0.89	13.00	42.60	26.37	10.62	0.53	1.03
	44,134	1.28	2.59	61.66	21.20	17.14	8.03	0.13	0.56	13.00	46.10	28.91	10.35	0.68	0.95
	60–30	-	-	59.66	17.20	23.14	8.09	0.18	0.33	13.50	44.70	26.06	12.17	0.80	0.73
	120–60	-	-	61.66	21.20	17.14	8.12	0.19	0.11	15.00	45.00	27.83	10.78	1.11	0.78
SYSW33	18–0	1.17	2.61	67.51	17.21	15.28	7.81	0.14	0.89	9.50	46.50	28.86	11.13	0.46	1.10
	50–18	1.27	2.6	65.51	15.21	19.28	7.88	0.12	0.45	10.50	46.70	30.40	10.60	0.53	0.61
	90–50	-	-	65.51	15.21	19.28	7.92	0.11	0.33	13.50	46.60	26.95	14.92	0.68	0.35
	100–90	-	-	61.51	17.21	21.28	7.93	0.12	0.11	17.50	45.80	27.64	13.07	0.97	0.37
SYSW34	18–0	1.18	2.61	63.58	19.43	16.99	7.91	0.13	1.00	17.00	45.20	27.64	12.20	1.03	1.18
	50–18	1.30	2.56	65.58	17.43	16.99	8.03	0.12	0.56	18.00	46.80	28.58	11.59	1.06	0.85
	80–50	–	–	65.58	17.43	16.99	8.11	0.18	0.33	18.50	47.20	26.62	13.79	1.42	0.60
	150–80	–	–	65.58	17.43	16.99	8.16	0.26	0.11	20.50	45.00	24.36	13.17	1.51	0.48
SYSW35	25–0	1.21	2.56	55.58	21.21	21.21	7.87	0.11	0.78	12.50	43.80	28.74	9.55	0.73	1.22
	50–25	1.29	2.54	59.58	21.21	19.21	7.91	0.13	0.45	13.00	44.80	28.82	9.25	0.87	1.06
	90–50	–	–	61.58	21.21	17.21	8.01	0.20	0.33	13.00	45.60	27.59	10.82	0.99	0.78
	180–90	–	–	59.58	21.21	19.21	8.09	0.24	0.11	14.50	44.90	26.55	11.75	1.37	0.67
	220–180	–	–	53.58	21.21	25.21	8.13	0.41	0.11	33.50	39.60	23.63	10.72	1.38	0.40
SYSW36	15–0	1.16	2.55	59.73	23.21	17.06	7.86	0.11	0.89	3.50	45.70	26.05	14.32	0.85	1.34
	50–15	1.28	2.57	63.73	19.21	17.06	7.96	0.09	0.56	4.50	49.20	25.58	17.73	0.94	0.95
	100–50	-	-	61.73	21.21	17.06	8.09	0.16	0.22	5.50	46.40	23.39	18.08	1.00	0.83
SYSW37	20–0	1.18	2.60	57.58	25.43	16.99	7.97	0.14	0.78	12.00	43.90	26.18	11.59	0.90	1.14
	60–20	1.26	2.53	61.58	19.43	18.99	8.08	0.17	0.45	13.00	45.50	25.52	13.97	1.16	0.86
	120–60	-	-	63.58	18.43	17.99	8.10	0.19	0.33	20.00	48.90	27.95	14.44	1.37	0.61
SYSW38	18–0	1.10	2.56	56.58	24.43	18.99	7.81	0.10	1.34	18.50	41.10	26.33	9.58	0.63	0.96
	50–18	1.31	2.55	58.58	22.43	18.99	7.95	0.14	0.78	19.50	42.40	27.48	9.80	0.73	0.75
	90–50	-	-	61.58	19.43	18.99	8.02	0.19	0.56	20.00	45.50	28.06	11.05	1.04	0.71
	130–90	-	-	61.58	21.43	16.99	8.09	0.29	0.22	24.00	45.60	27.32	12.45	1.25	0.54
	200–130	-	-	59.58	21.43	18.99	8.10	0.36	0.11	35.00	42.20	27.57	10.49	1.26	0.41
	260–200	-	-	51.58	21.43	26.99	8.13	0.35	0.11	38.50	37.70	22.62	10.77	1.45	0.45
SYS1	0–30	1.40	2.53	40.00	34.00	26.00	6.86	0.07	2.12	0.95	42.30	24.00	11.00	0.40	0.30
	30–60	1.50	2.54	46.00	28.00	26.00	7.04	0.05	1.92	0.97	43.10	27.00	10.00	0.60	0.20
	60–80	1.50	2.54	46.00	28.00	26.00	7.04	0.05	1.67	0.97	43.10	27.00	10.00	0.60	0.20
SYS2	0–20	1.10	2.54	40.00	32.00	28.00	7.26	0.07	2.04	0.95	42.30	27.00	12.00	0.40	0.30
	20–40	1.20	2.54	48.00	27.00	25.00	7.34	0.05	0.78	0.97	43.10	28.00	10.00	0.60	0.20
	40–80	1.20	2.54	48.00	27.00	25.00	7.34	0.05	0.78	0.97	43.10	28.00	10.00	0.60	0.20
SYS3	0–25	1.40	2.40	38.00	32.00	30.00	6.25	0.04	1.89	0.95	38.20	25.00	10.00	0.50	0.30
	25–50	1.45	2.50	40.00	36.00	24.00	7.30	0.04	0.81	0.95	38.40	26.00	9.00	0.30	0.50
	50–90	1.45	2.50	40.00	36.00	24.00	7.33	0.05	0.75	0.95	38.40	26.00	9.00	0.30	0.40
SYS4	0–30	1.40	2.45	32.00	38.00	22.00	7.20	0.05	1.01	0.43	38.20	25.00	10.00	0.70	0.50
	30–70	1.45	2.55	34.00	36.00	22.00	7.22	0.04	0.78	0.95	38.40	26.00	9.00	0.60	0.60
	70–90	1.45	2.55	34.00	36.00	22.00	7.25	0.04	0.23	0.95	38.40	26.00	9.00	0.60	0.70
SYS5	0–20	1.40	2.40	38.00	32.00	30.00	7.25	0.04	1.89	0.95	42.30	27.00	12.00	0.40	0.30
	20–50	1.45	2.50	40.00	36.00	24.00	7.30	0.04	0.81	0.95	43.10	28.00	10.00	0.60	0.20
	50–90	1.45	2.50	40.00	36.00	24.00	7.33	0.05	0.75	0.95	43.10	28.00	10.00	0.60	0.20
SYS6	0–20	1.40	2.54	50.00	28.00	22.00	7.30	0.05	2.04	0.95	44.10	29.00	10.00	0.40	1.50
	20–40	1.45	2.55	54.00	24.00	22.00	7.34	0.04	1.69	1.36	45.20	31.00	9.00	0.40	1.20
	40–100	1.45	2.55	54.00	24.00	22.00	7.35	0.04	1.60	1.55	45.20	31.00	9.00	0.40	1.60
SYS7	0–30	1.45	2,55	46.00	42.00	12.00	7.15	0.06	0.56	0.43	43.30	26.00	11.00	0.30	0.40
	30–70	1.50	2.60	46.00	36.00	18.00	7.25	0.06	0.45	0.95	43.40	26.00	10.00	0.30	0.20
	70–110	1.50	2.60	48.00	32.00	18.00	7.25	0.06	0.22	1.36	43.60	27.00	10.00	0.40	0.20
SYS8	0–20	1.40	2.50	40.00	30.00	30.00	7.20	0.05	1.12	0.95	42.10	27.00	10.00	0.50	1.70
	20–50	1.45	2.55	50.00	30.00	20.00	7.23	0.04	0.89	0.93	43.40	27.00	9.00	0.40	1.40
	50–110	1.45	2.55	54.00	24.00	22.00	7.23	0.03	0.78	1.46	43.40	28.00	9.00	0.40	1.10
SYS9	0–20	1.35	2.60	52.00	24.00	24.00	7.20	0.09	1.67	0.95	43.80	27.00	11.00	0.50	1.10
	20–50	1.45	2.62	54.00	22.00	24.00	7.24	0.07	1.23	1.33	44.30	28.00	10.00	0.50	0.90
	50–90	1.45	2.62	52.00	24.00	24.00	7.27	0.06	1.23	1.44	44.30	28.00	10.00	0.50	0.90
SYS10	0–25	1.35	2.60	50.00	30.00	30.00	7.25	0.06	2.56	0.93	44.10	28.00	11.00	0.40	0.30
	25–50	1.45	2.65	56.00	26.00	28.00	7.35	0.06	1.12	1.46	45.30	29.00	10.00	0.60	0.20
	50–100	1.45	2.65	56.00	26.00	28.00	7.35	0.06	1.12	1.46	45.30	29.00	10.00	0.60	0.20
SYS11	0–25	1.40	2.60	68.00	16.00	16.00	7.71	0.10	1.12	3.25	56.10	39.00	12.00	0.50	1.70
	25–60	1.50	2.65	66.00	18.00	16.00	7.75	0.12	0.92	4.46	56.10	40.00	10.00	0.60	1.70
	60–100	1.50	2.65	68.00	16.00	16.00	7.75	0.13	0.81	4.46	56.20	40.00	10.00	0.60	1.70
SYS12	0–20	1.40	2.65	58.00	20.00	32.00	7.71	0.07	1.01	1.46	44.60	31.00	9.00	0.40	0.30
	20–50	1.45	2.66	64.00	16.00	30.00	7.81	0.08	0.89	3.51	45.50	33.00	8.00	0.60	0.10
	50–100	1.45	2.66	64.00	16.00	30.00	7.81	0.08	0.89	3.51	45.60	33.00	8.00	0.60	0.10
SYS13	0–30	1.40	2.65	56.00	22.00	22.00	7.81	0.08	1.67	2.96	48.20	33.00	10.00	1.20	1.90
	30–60	1.50	2.66	60.00	20.00	20.00	7.83	0.08	0.98	3.46	49.40	34.00	9.00	1.20	1.80
	60–100	1.55	2.66	60.00	20.00	20.00	7.83	0.01	0.76	3.97	49.40	34.00	9.00	1.10	1.40
SYS14	0–20	1.40	2.60	60.00	26.00	14.00	7.80	0.08	1.67	3.93	55.20	38.00	11.00	0.90	1.90
	20–40	1.60	2.65	63.00	23.00	14.00	7.85	0.08	0.96	4.97	55.90	39.00	10.00	1.10	1.60
	40–110	1.60	2.65	64.00	23.00	13.00	7.85	0.08	0.67	4.97	56.10	39.00	10.00	1.10	1.60
SYS15	0–20	1.30	2.60	54.00	26.00	20.00	7.55	2.13	1.67	1.46	46.20	31.00	10.00	0.70	1.80
	20–40	1.35	2.62	60.00	20.00	20.00	7.60	3.09	1.12	1.75	47.10	34.00	9.00	0.60	1.60
	40–90	1.35	2.62	60.00	16.00	28.00	7.60	3.10	0.56	2.46	47.10	34.00	9.00	1.10	1.50
SYS16	0–20	1.40	2.66	40.00	42.00	18.00	7.51	0.09	1.01	1.46	37.20	25.00	9.00	1.20	1.90
	20–40	1.50	2.65	56.00	26.00	18.00	7.55	0.07	0.67	1.95	41.40	28.00	8.00	1.20	1.80
	40–100	1.55	2.65	60.00	24.00	16.00	7.55	0.07	0.22	2.45	41.70	29.00	8.00	1.10	1.40
SYS17	0–20	1.40	2.65	56.00	22.00	22.00	7.71	0.08	1.67	2.96	47.60	30.00	10.00	1.20	1.90
	20–50	1.50	2.66	58.00	20.00	22.00	7.73	0.08	0.78	3.46	49.90	34.00	9.00	1.20	1.80
	50–120	1.55	2.66	58.00	20.00	22.00	7.73	0.10	0.56	3.97	49.90	34.00	9.00	1.10	1.40
SYD1	20–0	1.27	2.58	66.70	14.50	18.80	7.26	0.24	1.37	0.21	50.26	25.81	13.25	1.48	1.27
	60–20	1.27	2.59	66.90	16.20	16.90	7.27	0.19	0.64	1.11	52.77	30.13	12.87	1.62	1.15
	100–60	1.28	2.61	68.90	16.30	14.80	7.27	0.15	0.30	1.49	52.42	31.27	13.61	1.14	1.10
SYD2	20–0	1.28	2.59	65.90	16.80	17.30	7.23	0.20	1.40	0.52	50.67	25.58	13.74	1.50	1.18
	60–20	1.28	2.59	67.40	15.80	16.80	7.24	0.15	0.61	0.85	53.28	28.08	15.37	1.84	0.96
	100–60	1.29	2.61	67.60	15.90	16.50	7.26	0.15	0.41	1.93	52.72	29.35	15.70	1.50	0.92
SYD3	15–0	1.26	2.59	64.80	19.50	15.70	7.25	0.14	1.22	0.95	49.23	24.91	13.40	1.62	1.23
	60–15	1.27	2.61	66.30	17.80	15.90	7.27	0.23	0.51	1.73	52.83	27.76	14.85	2.16	1.14
	110–60	1.29	2.61	67.10	15.50	17.40	7.27	0.19	0.27	1.77	51.39	28.69	14.22	2.29	0.98
SYD4	18–0	1.27	2.59	64.10	20.60	15.30	7.28	0.21	1.31	1.16	47.73	23.49	12.56	2.13	1.22
	60–18	1.28	2.60	64.80	18.90	16.30	7.28	0.21	0.70	1.65	48.39	24.12	13.85	2.38	1.17
	110–60	1.28	2.60	65.20	20.90	13.90	7.30	0.13	0.28	2.41	48.91	26.62	13.26	2.33	1.05
SYD5	20–0	1.26	2.60	65.30	20.20	14.50	7.28	0.16	1.28	1.70	47.52	25.38	11.60	1.73	1.19
	60–20	1.28	2.60	65.50	17.90	16.60	7.29	0.13	0.55	2.29	48.27	25.75	13.22	1.90	1.15
SYD6	20–0	1.26	2.59	64.10	20.80	15.10	7.23	0.12	1.38	0.78	47.91	25. 23	12.18	1.60	1.16
	55–20	1.28	2.61	65.10	20.30	14.60	7.23	0.22	0.68	1.25	49.35	26.81	13.41	1.67	0.96
	80–55	1.29	2.61	65.50	18.40	16.10	7.24	0.17	0.41	1.66	50.67	29.08	13.56	1.98	0.78
SYD7	0–12	1.26	2.58	60.60	22.10	17.30	7.66	0.38	1.39	4.94	46.28	24.69	11.87	1.76	1.19
	40–12	1.27	2.60	61.90	20.80	17.30	7.66	0.53	0.66	8.22	47.77	24.82	13.68	1.92	1.28
	85–40	1.29	2.60	64.20	20.50	15.30	7.68	0.51	0.25	11.69	47.10	28.11	13.22	1.69	0.80
	130–85	1.30	2.61	64.80	20.50	14.70	7.70	0.33	0.22	11.92	49.37	30.29	13.25	1.28	0.64
SYD8	0–10	1.26	2.58	61.50	20.80	17.70	7.29	0.38	1.14	3.52	45.66	23.14	11.78	2.23	1.27
	38–10	1.27	2.58	61.90	21.60	16.50	7.29	0.41	0.63	6.13	45.98	24.55	12.31	1.87	0.91
	80–38	1.29	2.60	64.40	19.10	16.50	7.29	0.53	0.27	8.91	47.73	26.83	12.69	1.17	0.77
	125–80	–	–	63.60	20.30	16.10	7.31	0.29	–	8.94	47.20	29.15	12.45	1.08	0.62
SYD9	30–0	1.27	2.58	59.30	20.50	20.20	7.29	0.76	1.13	5.93	43.62	22.94	11.18	1.53	1.20
	60–30	1.29	2.59	61.60	18.20	20.20	7.30	0.55	0.48	8.72	44.28	24.28	11.62	1.84	1.16
	100–60	1.29	2.61	63.40	18.20	18.40	7.30	0.59	0.21	13.57	45.96	26.16	13.30	1.23	0.89
	150–100	–	–	63.40	17.40	19.20	7.32	0.19	–	13.28	45.51	27.39	13.69	1.17	0.76
SYD10	22–0	1.27	2.59	58.20	20.10	21.70	7.33	0.44	1.59	4.91	44.47	22.61	12.59	1.67	0.98
	55–22	1.28	2.60	61.10	20.00	18.90	7.33	0.38	0.68	7.28	47.15	25.81	12.52	2.13	0.83
	100–55	1.28	2.60	63.20	19.90	16.90	7.35	0.37	0.29	9.55	49.28	27.16	14.83	1.93	0.79
	140–100	1.30	2.61	63.80	20.10	16.10	7.36	0.13	0.21	9.78	49.83	28.12	14.27	1.90	0.72
SYD11	25–0	1.27	2.58	60.90	18.20	20.90	7.39	0.36	1.02	4.11	43.59	23.61	10.56	1.94	1.28
	50–25	1.27	2.58	62.90	17.90	19.20	7.41	0.45	0.47	4.69	45.52	23.89	12.68	2.43	0.91
	100–50	1.29	2.58	63.20	17.50	19.30	7.43	0.42	0.22	8.19	47.85	26.72	13.16	2.27	0.78
	130–100	–	–	62.80	20.50	17.70	7.43	0.29	–	11.28	47.36	26.95	13.67	1.65	0.73
SYD12	20–0	1.27	2.58	56.20	22.60	21.20	7.44	0.68	0.98	5.60	43.38	22.34	11.35	1.66	0.92
	85–20	1.29	2.58	59.20	21.00	19.80	7.47	0.82	0.52	7.69	45.13	24.40	11.94	1.81	0.95
	110–85	1.29	2.60	59.40	21.30	19.30	7.47	0.73	0.29	10.83	44.27	24.82	12.55	1.87	0.61
SYD13	0–10	1.26	2.58	56.90	20.30	22.80	7.45	0.61	1.07	7.22	43.28	23.56	10.28	1.93	0.99
	55–10	1.28	2.59	59.50	19.60	20.90	7.47	0.87	0.67	10.56	45.91	24.87	12.45	1.69	0.71
	55–120	1.29	2.59	59.80	19.80	20.40	7.47	0.59	0.23	12.73	46.78	26.37	13.49	1.80	0.87
SYD14	25–0	1.27	2.57	56.40	22.30	21.30	7.63	0.41	1.29	4.19	44.16	23.79	11.34	1.31	1.13
	70–25	1.27	2.60	59.30	22.60	18.10	7.65	0.47	0.53	9.76	46.65	25.25	12.63	1.76	0.98
	120–70	1.29	2.60	61.30	22.60	16.10	7.66	0.38	0.26	11.85	46.78	26.90	12.88	1.79	0.76
SYD15	0–10	1.26	2.57	56.30	21.20	22.50	7.71	0.65	1.18	8.24	43.38	23.36	10.23	2.31	1.15
	50–10	1.26	2.58	58.40	20.50	21.10	7.71	0.72	0.59	8.69	43.95	24.02	10.92	2.39	0.89
	100–50	1.29	2.58	60.30	20.10	19.60	7.74	0.21	0.24	12.54	45.76	24.89	13.70	1.27	0.75
SYD16	10–0	1.28	2.58	55.60	22.10	22.30	7.77	0.47	1.13	6.89	43.12	22.69	11.27	1.84	1.23
	45–10	1.28	2.59	56.80	22.60	20.60	7.79	0.63	0.60	12.33	45. 87	24.48	11.91	2.11	1.16
	70–45	1.29	2.59	59.90	20.20	19.90	7.79	0.41	0.27	13.94	45.92	24.97	12.33	1.95	0.84
	120–70	1.30	2.60	58.60	21.20	20.20	7.81	0.20	0.23	15.12	46.48	25.89	13.80	1.37	0.64
SYD17	0–12	1.26	2.58	57.30	20.80	21.90	7.79	0.39	1.39	9.61	42.78	22.62	10.28	2.21	1.39
	50–12	1.28	2.59	57.80	21.90	20.30	7.79	0.47	0.71	11.73	44.14	23.30	11.61	2.34	1.17
	80–50	1.28	2.59	61.20	20.30	18.50	7.79	0.45	0.32	17.76	45.67	25.72	11.69	1.96	0.81
	135–80	–	–	60.80	20.50	18.70	7.81	0.26	–	20.94	45.22	25.49	13.48	1.15	0.74
SYD18	0–10	1.27	2.59	55.80	21.10	23.10	7.81	0.24	1.09	4.97	42.25	21.38	11.43	1.89	1.27
	40–10	1.29	2.61	58.90	20.20	20.90	7.81	0.51	0.61	9.17	44.97	23.45	11.79	2.23	0.90
	110–40	1.29	2.61	57.60	20.90	21.50	7.82	0.37	0.21	10.85	45.20	24.78	12.20	2.16	0.81
SYD19	30–0	1.27	2.58	54.40	23.90	21.70	7.88	0.43	1.45	9.39	40.42	20.22	11.81	1.60	1.12
	90–30	1.27	2.60	57.10	22.50	20.40	7.88	0.62	0.49	19.25	44.26	23.61	12.55	1.82	0.86
	140–90	1.29	2.60	60.90	20.90	18.20	7.89	0.59	0.25	23.90	45.53	24.46	14.38	1.27	0.61
SYD20	0–10	1.26	2.58	57.20	20.50	22.30	7.81	0.49	1.17	8.68	43.39	22.57	10.28	2.63	1.12
	50–10	1.27	2.58	59.10	20.20	20.70	7.81	0.67	0.59	11.76	44.27	24.35	10.66	2.51	0.87
	110 - 50	1.29	2.61	60.70	19.70	19.60	7.82	0.61	0.20	11.92	46.71	25.77	13.50	1.93	0.84
SYD21	0–12	1.28	2.58	56.70	20.40	22.90	7.81	0.49	1.12	9.23	43.52	22.59	10.57	2.44	1.23
	50 - 12	1.28	2.59	59.60	19.70	20.70	7.85	0.67	0.64	12.76	45.88	24.83	11.63	1.95	1.18
	120–50	1.30	2.59	59.90	18.20	21.90	7.85	0.75	0.26	13.92	45.36	25.81	11.97	1.49	0.89
SYD22	0–12	1.27	2.59	54.80	22.00	23.20	7.89	0.45	1.27	7.84	42.47	22.39	10.15	2.36	1.41
	60- 12	1.29	2.61	56.70	21.50	21.80	7.91	0.83	0.61	12.67	44.79	24.92	10.37	2.48	1.16
	90 −60	1.30	2.61	59.20	19.50	21.30	7.91	0.69	0.33	16.23	47.24	27.63	12.49	2.13	0.81
	130- 90	–	–	60.60	20.60	18.80	7.94	0.54	–	15.12	47.66	27.68	13.42	1.83	0.72
SYD23	32–0	1.28	2.60	57.50	20.70	21.80	7.78	0.88	1.29	6.27	42.61	21.72	11.19	1.95	1.21
	75–32	1.29	2.60	60.30	20.10	19.60	7.79	0.96	0.68	9.43	47.46	25.50	12.23	2.41	0.91
	120–75	1.29	2.61	62.50	20.50	17.00	7.79	0.71	0.29	10.57	47.70	27.56	12.54	2.69	0.78
SYD24	30–0	1.27	2.57	55.40	20.70	23.90	7.71	0.85	1.67	5.22	43.58	23.69	10.45	1.76	1.13
	65–30	1.29	2.57	59.90	18.20	21.90	7.75	0.93	0.71	10.12	46.37	25.11	12.57	1.88	1.03
	110–65	1.30	2.58	61.20	18.50	20.30	7.76	0.74	0.37	13.64	46.65	25.16	13.72	2.21	0.89
SYD25	28–0	1.27	2.58	56.10	20.80	23.10	7.75	0.97	1.73	7.98	42.73	22.38	11.43	1.85	1.22
	60–28	1.28	2.59	60.40	19.70	20.90	7.77	0.90	0.59	10.36	48.76	26.59	12.28	2.74	0.96
	110–60	1.28	2.59	61.70	19.10	19.20	7.77	0.82	0.38	11.57	47.91	28.11	13.68	1.59	0.82
SYD26	10–00	1.27	2.56	57.60	19.90	22.50	7.95	0.73	1.18	5.38	42.35	22.46	10.65	1.92	1.63
	50–10	1.29	2.56	58.60	19.30	22.10	7.97	0.55	0.53	9.42	43.93	24.30	10.71	1.98	1.25
	110–50	1.30	2.58	60.80	19.80	19.40	7.96	0.52	0.22	12.94	45.12	25.93	12.67	1.79	1.14
SYD27	20–0	1.28	2.57	51.30	22.90	25.80	7.88	0.87	0.92	6.13	36.45	17.57	10.93	1.80	0.96
	50–20	1.30	2.57	54.90	22.60	22.50	7.89	0.89	0.43	6.28	37.28	17.85	11.59	2.17	0.93
	100–50	1.31	2.58	58.70	21.90	19.40	7.95	0.66	0.21	11.81	42.30	22.81	12.27	1.53	0.79
	150–100	–	–	61.40	19.50	19.10	7.95	0.53	–	12.97	42.87	23.63	12.50	1.47	0.72
SYD28	18–0	1.27	2.57	51.30	23.80	24.90	7.81	0.84	0.99	6.92	35.52	17.49	9.89	1.71	1.28
	50–18	1.28	2.57	51.90	24.90	23.20	7.82	0.71	0.46	7.96	35.76	18.25	10.16	1.90	0.81
	80–50	1.30	2.57	55.90	22.60	21.50	7.82	0.66	0.22	10.67	40.39	20.74	13.58	1.56	0.81
	140–80	–	–	55.20	24.00	20.80	7.82	0.39	–	12.31	39.71	20.14	13.70	1.23	0.67
SYD29	25–0	1.28	2.58	49.10	25.10	25.80	7.87	0.71	0.93	7.82	35.27	17.19	9.61	2.21	1.08
	60–25	1.30	2.59	51.40	24.30	24.30	7.89	0.77	0.41	9.56	36.51	18.25	10.16	2.48	0.93
	90–60	1.30	2.59	54.50	22.90	22.60	7.91	0.65	0.23	10.59	40.78	20.92	12.45	1.82	0.75
	130–90	–	–	55.50	22.70	21.80	7.91	0.40	–	13.25	40.22	20.21	13.34	1.82	0.68
SYD30	22–0	1.28	2.57	46.60	25.90	27.50	7.92	0.66	0.98	6.28	40.86	22.36	10.32	1.57	0.89
	60–22	1.29	2.58	50.70	23.50	25.80	7.92	0.71	0.52	11.54	44.12	23.46	12.51	1.80	0.97
	100–60	1.31	2.60	49.10	23.40	27.50	7.94	0.43	0.27	13.37	45.36	26.11	11.56	1.84	0.93
SYD31	33 –0	1.28	2.57	47.90	25.50	26.60	7.97	0.84	1.13	8.92	40.81	22.57	10.16	1.74	0.93
	105–33	1.30	2.58	49.60	25.50	24.90	7.99	0.95	0.44	9.74	44.70	23.76	11.85	2.52	1.10
	160–105	1.31	2.58	50. 7	24.10	25.20	7.99	0.93	0.23	12.61	44.59	25.39	11.77	1.83	0.88
SYD32	20–0	1.28	2.57	40.70	27.70	31.60	7.93	0.43	0.88	12.75	30.63	14.47	8.59	2.19	1.16
	50–20	1.28	2.59	43.40	28.00	28.60	7.93	0.45	0.36	16.83	32.25	16.24	9.13	1.92	1.11
	100–50	1.29	2.59	44.00	28.90	27.10	7.93	0.32	0.19	21.96	35.59	17.11	11.87	1.65	0.93
	140–100	–	–	46.80	25.80	27.40	7.94	0.28	–	22.37	32.38	15.85	10.56	1.62	0.76
SYD33	30–0	1.28	2.57	40.80	28.60	30.60	7.76	0.49	0.72	12.16	28.96	14.10	8.19	1.88	0.99
	60–30	1.28	2.59	43.70	28.10	28.20	7.76	0.81	0.28	15.74	31.55	15.49	9.87	1.36	1.04
	100–60	1.30	2.59	47.20	26.90	25.90	7.77	0.42	0.19	21.35	31.89	15.72	10.35	1.25	0.84
	140–100	–	–	47.80	26.00	26.20	7.79	0.28	–	24.93	30.38	15.54	10.13	0.95	0.71
SYD34	18–0	1.28	2.58	40.50	29.60	29.90	7.96	0.91	0.71	10.72	28.20	14.31	7.26	1.86	1.15
	70–18	1.29	2.59	44.20	28.80	27.00	7.97	0.98	0.39	16.57	31.52	14.63	10.73	1.49	1.15
	130–70	1.31	2.59	45.80	27.50	26.70	7.97	0.56	0.20	18.33	31.66	15.32	10.87	1.35	0.88
SYD35	30–0	1.27	2.58	41.70	27.50	30.80	8.11	0.34	0.78	12.97	29.75	15.21	7.72	1.83	1.17
	100–30	1.29	2.58	44.90	27.30	27.80	8.11	0.45	0.30	16.15	32.91	15.59	10.32	2.12	1.17
	160–100	1.30	2.58	45.20	27.30	27.50	8.13	0.49	0.21	17.34	29.78	15.36	9.27	1.15	0.84
SYD36	20–0	1.29	2.56	37.80	30.40	31.80	8.14	0.49	0.70	15.74	28.10	14.38	7.15	1.86	0.95
	20–70	1.31	2.58	30.70	30.60	38.70	8.15	0.97	0.39	23.89	24.51	12.56	6.63	1.27	1.13
SYD37	20–0	1.29	2.57	40.50	28.90	30.60	8.22	1.17	0.40	23.41	24.97	12.25	7.17	1.42	0.88
	20+	Rocks													
SYD38	30–0	1.30	2.56	38.00	30.40	31.60	8.17	0.81	0.61	14.27	27.06	12.52	8.28	1.51	1.10
	80–30	1.30	2.56	41.80	30.00	28.20	8.19	0.93	0.27	17.98	34.61	17.39	10.43	1.67	0.75
	120–80	1.31	2.57	44.10	29.80	26.10	8.20	0.90	0.20	21.67	33.12	17.61	9.27	1.73	0.72
SYD39	20–0	1.29	2.58	38.30	27.90	33.80	8.15	0.41	0.54	14.73	25.96	12.19	7.36	1.70	1.14
	50–20	1.30	2.58	44.60	25.10	30.30	8.17	0.49	0.22	21.42	30.41	15.68	8.18	1.88	1.11
	110–50	1.30	2.60	43.90	25.90	30.20	8.17	0.37	0.18	22.31	31.84	15.65	10.70	1.73	0.66
SYD40	30–0	1.29	2.55	30.30	39.30	30.40	8.11	0.23	0.59	16.93	23.75	10.32	7.60	1.68	1.10
	60–30	1.29	2.56	35.50	37.80	26.70	8.13	0.26	0.31	18.29	28.61	13.42	9.36	1.41	1.18
	100–60	1.31	2.58	38.20	36.60	25.20	8.13	0.71	0.18	22.59	27.79	12.95	9.86	1.77	0.84
	140–100	–	–	39.70	33.60	26.70	8.13	0.32	–	23.26	24.54	11.82	8.51	1.30	0.65
SYD41	0–12	1.29	2.57	34.50	30.70	34.80	8.19	0.87	0.39	19.53	24.56	12.33	6.21	1.77	1.12
	12 <	Rocks													
SYD42	20–0	1.29	2.54	32.20	34.50	33.30	8.10	0.53	0.52	19.42	24.57	12.34	6.39	1.55	1.10
	50–20	1.30	2.55	34.10	34.70	31.20	8.12	0.55	0.36	23.86	25.38	12.47	6.92	1.70	1.13
	80–50	1.32	2.55	32.70	36.80	30.50	8.12	0.61	0.19	29.77	28.91	15.02	8.25	1.33	0.96
	120–80	–	–	29.90	37.80	32.30	8.13	0.58	–	32.46	26.14	13.97	7.15	1.30	0.89
SYD43	25–0	1.30	2.55	31.30	38.20	30.50	8.11	0.54	0.47	31.25	23.87	12.08	6.53	1.45	0.96
	25 <	Rocks													
SYD44	15–0	1.29	2.57	40.70	29.10	30.20	8.12	0.83	0.49	19.17	24.46	11.57	7.44	1.27	0.98
	40–15	1.30	2.57	46.40	25.90	27.70	8.14	0.92	0.28	26.25	28.72	14.37	8.19	1.61	0.87
	80–40	1.31	2.59	46.90	25.90	27.20	8.14	0.41	0.22	26.34	31.13	16.59	8.54	1.40	0.87
	120–80	–	–	43.80	25.80	30.40	8.14	0.09	–	24.15	28.83	15.25	8.81	1.32	0.79
SYD45	25–0	1.30	2.56	36.70	33.20	30.10	8.11	0.61	0.56	26.67	24.63	12.39	7.18	1.33	0.91
	< 25	ROCK													
SYD46	20–0	1.29	2.55	37.40	35.30	27.30	8.12	0.67	0.51	17.35	26.39	12.85	7.26	1.72	1.15
	75–20	1.29	2.57	40.00	32.80	27.20	8.12	0.62	0.32	21.12	29.13	14.48	8.10	1.87	1.11
	115–75	1.31	2.57	36.80	36.20	27.00	8.13	0.49	0.24	28.86	30.71	16.22	8.49	1.57	0.91
	140–115	–	–	32.10	33.80	34.10	8.15	0.36	–	29.42	30.18	16.39	8.23	1.30	0.87
SYD47	25–0	1.29	2.57	39.30	30.80	29.90	8.12	0.85	0.51	12.31	23.48	11.36	6.30	1.68	1.10
	65–25	1.31	2.58	43.80	30.00	26.20	8.14	0.90	0.24	17.12	28.79	14.51	7.93	1.80	1.12
	100–65	1.31	2.58	43.50	30.10	26.40	8.14	0.62	0.21	18.64	27.94	14.88	7.47	1.65	0.92

BD = Bulk density, PD = practical density, Sn = sand, *C* = clay, *S*= silty, *L*= loam, EC = Electrical conductivity, TOM = Total organic matter, CEC = Cation exchange capacity by 1 M NH_4_OAc (pH = 7.0).

**Table 4:** Macro and micro nutrients in some representative soil profiles in the southern region of Syria.

Profile	Horizon cm	N	P	K	Cu	Fe	Mn	Zn	B
		% mg/kg							
SYDJ1	0- 20	9.20	1.83	–	–	–	–	–	–
	20–55	10.50	1.83	–	1.42	31.50	29.50	1.40	0.53
	55–89	12.20	1.40	–	1.50	26.60	30.70	1.23	0.50
SYDJ2	0–30	10.50	4.50	–	4.15	75.52	73.60	1.94	0.50
SYDJ3	0–10	9.00	4.80	–	2.53	18.80	12.40	0.82	0.15
	10–50	12.00	1.83	–	–	–	–	–	–
	50–90	10.00	1.41	–	1.42	12.30	13.64	0.50	0.10
	90	8.00	1.90	–	–	–	–	–	–
SYDJ4	0–15	14.00	5.20	–	0.80	13.04	8.90	0.32	0.45
	15–55	12.00	5.20	–	0.63	10.82	9.60	0.39	0.23
SYDJ5	0–20	9.20	8.60	–	2.13	38.60	24.10	0.96	0.67
SYSW1	18–0	0.11	49.80	–	3.14	25.61	24.35	2.98	0.10
	40–18	0.10	28.00	–	2.65	25.39	23.79	1.51	0.16
	100–40	-	-	–	-	-	-	-	-
SYSW2	22–0	0.28	41.10	–	1.27	35.13	24.79	3.59	0.17
	45–22	0.13	16.40	–	2.26	34.91	27.67	3.37	0.25
	75–45	-	-	–	-	-	-	-	-
SYSW3	25–0	0.15	36.30	–	2.28	22.39	45.90	3.01	0.37
	90–25	0.05	32.80	–	2.51	25.95	16.52	1.68	0.09
SYSW4	22–0	0.03	94.80	–	0.70	12.45	4.12	5.20	0.14
	50–22	0.05	38.20	–	2.15	15.13	9.72	3.60	0.22
	85–50	-	-	–	-	-	-	-	-
SYSW5	12–0	0.10	11.30	–	1.51	17.36	30.71	5.25	0.16
	50–12	0.10	6.60	–	1.73	20.35	20.64	7.73	0.19
SYSW6	18–0	0.09	60.90	–	2.27	15.03	8.42	3.90	0.13
	60–18	0.08	48.90	–	1.53	10.01	2.30	5.96	0.11
	90–60	-	-	–	-	-	-	-	-
SYSW7	17–0	0.12	54.40	–	3.12	31.30	33.49	4.82	0.10
	30–17	0.07	37.30	–	2.12	21.35	17.27	6.81	0.11
	50–30	–	–	–	–	–	–	–	–
	110–50	–	–	–	–	–	–	–	–
SYSW8	12–0	0.10	11.30	–	1.51	17.36	30.71	5.25	0.16
	50–12	0.10	6.60	–	1.73	20.35	20.64	7.73	0.19
SYSW9	22–0	0.08	10.10	–	2.48	15.83	11.92	4.24	0.11
	35–22	0.07	4.60	–	2.41	12.49	5.12	4.45	0.17
	60–35	-	-	–	-	-	–	-	-
SYSW10	25–0	0.10	41.10	–	2.87	20.13	10.79	3.61	0.13
	40–25	0.09	16.40	–	2.26	18.30	14.67	3.40	0.11
	90–40	-	-	–	-	-	-	-	–
SYSW11	25–0	0.11	61.00	–	2.65	17.91	21.04	2.66	0.28
	80–25	0.08	35.40	–	2.36	27.70	15.37	4.59	0.42
	100–80	-	-	–	-	-	-	-	-
SYSW12	18–0	0.15	9.30	–	1.99	21.26	41.93	6.06	0.16
	55–18	0.09	1.50	–	2.26	18.27	17.19	6.59	0.24
	120–55	-	–	–	-	-	-	-	-
SYSW13	20–0	0.05	1.90	–	1.81	8.59	6.70	1.47	0.29
	45–20	0.05	1.20	–	1.32	6.16	1.87	2.35	0.35
	70–45	-	-	–	-	-	-	-	-
SYSW14	18–0	0.06	1.80	–	1.88	9.31	5.70	2.67	0.23
	40–18	0.04	0.90	–	0.95	5.01	1.45	1.96	0.32
	65–40	–	–	–	–	–	–	–	–
	100–65	–	–	–	–	–	–	–	–
SYSW15	18–0	0.05	3.20	–	1.88	9.31	5.70	2.67	0.17
	50–18	0.04	2.00	–	0.95	5.01	1.45	1.96	0.22
	90–50	-	–	–	–	–	–	–	–
SYSW16	18–0	0.08	20.50	–	2.94	29.28	55.95	2.96	0.04
	35–18	0.07	18.20	–	2.98	27.09	34.34	2.87	0.07
	70–35	-	-	–	-	-	-	-	-
	100–70	-	-	–	-	-	-	-	-
SYSW17	25–0	0.07	3.70	–	1.83	13.61	4.98	2.89	0.02
	50–25	0.08	2.00	–	1.98	14.57	8.00	2.83	0.04
	80–50	-	-	–	-	-	-	–	-
SYSW18	15–0	0.05	3.20	–	1.61	10.01	7.23	5.00	0.32
	40–15	0.04	1.40	–	1.57	5.94	3.39	6.12	0.11
	85–40	–	–	–	–	–	–	–	–
	110–85	–	–	–	–	–	–	–	–
SYSW19	20–0	0.11	9.80	–	2.20	14.74	20.28	4.66	0.22
	50–20	0.04	6.50	–	2.24	13.45	3.77	3.60	0.18
	100–50	-	-	–	-	-	-	-	-
SYSW20	18–0	0.05	3.20	–	2.31	14.18	24.27	4.00	0.24
	50–18	0.05	1.30	–	1.05	8.16	8.40	4.29	0.13
	110–50	-	-	–	-	-	-	-	-
SYSW21	14–0	0.05	5.30	–	1.72	9.14	9.87	2.06	0.10
	50–14	0.05	1.60	–	1.34	5.29	1.88	1.25	0.16
	70–50	–	–	–	–	–	–	–	–
	95–70	–	–	–	–	–	–	–	–
SYSW22	15–0	0.04	2.40	–	2.19	8.11	5.87	6.11	0.09
	60–15	0.05	1.00	–	1.16	6.16	2.85	4.57	0.11
	110–60	-	-	–	-	-	-	-	-
SYSW23	22–0	0.05	2.20	–	1.34	3.50	1.83	1.39	0.17
	60–22	0.05	1.70	–	1.48	3.17	1.96	10.04	0.12
	95–60	-	-	–	-	-	-	-	-
SYSW24	0–15	0.05	2.40	–	2.19	8.11	5.87	6.11	0.09
	15–60	0.05	1.00	–	1.16	6.16	2.85	4.57	0.11
	60–110	-	–	–	–	–	–	–	–
SYSW25	0–20	0.05	3.10	–	1.96	9.89	5.90	2.89	0.16
	20–50	0.05	1.80	–	1.03	5.45	2.01	2.01	0.20
	50–100	-	–	–	–	–	–	–	–
SYSW26	16–0	0.07	5.10	–	1.67	17.88	7.45	4.70	0.04
	40–16	0.06	2.70	–	1.64	12.47	2.89	4.50	0.05
	80–40	–	–	–	–	–	–	–	–
	100–80	–	–	–	–	–	–	–	–
SYSW27	0–10	0.07	2.60	–	2.00	9.90	6.00	3.00	0.12
	10–50	0.06	1.20	–	1.12	5.70	2.50	2.20	0.12
	90–50	-	–	–	–	–	–	–	–
SYSW28	18–0	0.03	1.70	–	1.65	8.42	5.28	6.46	0.12
	90–18	0.02	0.90	–	1.11	8.17	3.74	4.82	0.17
	120–90	-	–	–	-	-	-	-	-
SYSW29	15–0	0.07	14.70	–	1.90	4.29	3.80	6.12	0.11
	50–15	0.04	3.10	–	1.90	8.14	7.95	10.22	0.19
	130–50	-	–	–	-	-	-	-	-
SYSW30	20–0	0.04	6.40	–	2.14	14.11	9.73	6.48	0.06
	55–20	0.04	1.60	–	1.80	8.30	3.65	5.06	0.19
	100–55	-	–	–	-	-	-	-	-
SYSW31	15–0	0.04	4.80	–	2.41	12.94	6.72	6.46	0.09
	60–15	0.03	1.20	–	1.42	9.09	3.48	6.03	0.14
	110–60	-	–	–	-	-	-	-	-
SYSW32	0–10	0.07	2.30	–	1.43	5.45	8.71	6.05	0.05
	30-Oct	0.04	1.20	–	2.02	13.72	8.30	3.71	0.07
	60–30	-	-	–	-	-	-	-	-
	120–60	-	-	–	-	-	-	-	-
SYSW33	18–0	0.06	6.20	–	2.00	14.54	12.32	4.97	0.08
	50–18	0.05	1.40	–	1.33	5.71	1.97	4.30	0.10
	90–50	-	-	–	-	-	-	-	-
	100–90	-	-	–	-	-	-	-	-
SYSW34	18–0	0.05	5.80	–	2.25	8.69	6.48	7.69	0.20
	50–18	0.03	2.00	–	1.60	5.99	2.59	6.69	0.35
	80–50	–	–	–	–	–	–	–	–
	150–80	–	–	–	–	–	–	–	–
SYSW35	25–0	0.05	3.60	–	1.00	7.10	5.34	4.49	0.11
	50–25	0.04	1.40	–	0.66	3.32	3.52	3.19	0.11
	90–50	–	–	–	–	–	–	–	–
	180–90	–	–	–	–	–	–	–	–
	220–180	–	–	–	–	–	–	–	–
SYSW36	15–0	0.06	6.70	–	2.08	10.62	8.35	4.53	0.27
	50–15	0.05	1.30	–	2.34	8.87	3.17	9.11	0.29
	100–50	-	–	–	-	-	-	-	-
SYSW37	20–0	0.05	2.70	–	1.95	8.16	8.70	3.86	0.20
	60–20	0.04	0.60	–	1.77	7.86	5.27	4.85	0.23
	120–60	-	-	–	-	-	-	-	-
SYSW38	18–0	0.04	2.10	–	2.03	8.08	14.65	2.53	0.17
	50–18	0.04	1.30	–	1.24	5.01	8.43	4.73	0.24
	90–50	-	-	–	-	-	-	-	-
	130–90	-	-	–	-	-	-	-	-
	200–130	-	-	–	-	-	-	-	-
	260–200	-	-	–	-	-	-	-	-
SYS1	0–30	23.00	27.00	130.00	0.79	21.00	11.00	1.37	1.75
	30–60	17.00	20.00	120.00	0.75	16.00	10.80	1.08	1.70
	60–80	16.00	17.00	125.00	0.75	16.00	10.80	1.08	0.70
SYS2	0–20	20.00	23.00	140.00	1.51	16.00	17.00	1.37	1.22
	20–40	15.00	18.00	110.00	0.96	14.00	15.80	1.08	0.94
	40–80	13.00	15.00	115.00	0.96	13.00	15.80	1.08	0.94
SYS3	0–25	20.00	28.00	180.00	0.86	17.00	9.90	1.75	1.76
	25–50	14.00	15.00	130.00	0.85	15.00	9.25	1.82	0.66
	50–90	14.00	15.00	115.00	0.95	15.00	9.25	1.82	0.66
SYS4	0–30	15.00	20.00	170.00	0.19	18.00	2.32	1.22	0.13
	30–70	17.00	19.00	140.00	0.22	15.00	2.47	1.51	0.20
	70–90	16.00	11.00	110.00	0.27	14.00	2.35	1.48	0.10
SYS5	0–20	20.00	23.00	140.00	1.51	16.00	17.00	1.37	1.22
	20–50	15.00	18.00	110.00	0.95	14.00	15.80	1.08	0.94
	50–90	13.00	15.00	115.00	0.95	13.00	15.80	1.08	0.94
SYS6	0–20	20.00	20.00	250.00	0.90	19.00	21.05	2.56	1.10
	20–40	14.00	15.00	190.00	0.51	16.00	21.06	1.33	1.08
	40–100	14.00	12.00	150.00	0.49	12.00	20.75	1.46	1.03
SYS7	0–30	19.00	19.00	260.00	0.33	16.00	31.00	1.54	0.83
	30–70	13.00	17.00	190.00	0.14	15.00	20.05	1.44	0.70
	70–110	12.00	14.00	140.00	0.05	11.00	31.90	1.32	1.20
SYS8	0–20	21.00	25.00	260.00	0.06	17.00	8.30	2.42	0.16
	20–50	15.00	16.00	210.00	0.10	17.00	10.75	2.00	0.17
	50–110	13.00	14.00	230.00	1.00	13.00	9.90	2.11	0.25
SYS9	0–20	18.00	21.00	250.00	1.90	18.00	35.95	1.76	0.94
	20–50	13.00	21.00	200.00	2.07	16.00	35.95	1.51	0.63
	50–90	12.00	15.00	140.00	2.07	11.00	35.95	1.51	0.62
SYS10	0–25	15.00	20.00	240.00	0.56	12.00	32.05	3.20	0.94
	25–50	11.00	11.00	290.00	0.82	7.00	10.32	1.11	0.94
	50–100	10.00	11.00	250.00	0.82	6.00	10.32	1.11	0.94
SYS11	0–25	14.00	19.00	325.00	0.43	11.00	9.95	1.18	1.35
	25–60	10.00	10.00	280.00	0.65	6.00	10.25	1.32	1.76
	60–100	9.00	10.00	240.00	0.65	5.00	190.25	1.32	1.76
SYS12	0–20	12.00	15.00	320.00	0.44	10.00	7.70	1.14	0.77
	20–50	9.00	9.00	280.00	0.18	7.00	5.40	1.20	0.60
	50–100	7.00	9.00	230.00	0.18	5.00	5.50	1.20	0.60
SYS13	0–30	13.00	17.00	340.00	0.39	10.00	11.00	1.57	0.70
	30–60	9.00	9.00	290.00	0.25	7.00	10.00	1.54	0.60
	60–100	8.00	9.00	240.00	0.12	5.00	8.00	1.36	0.55
SYS14	0–20	12.00	15.00	375.00	0.45	10.00	9.25	1.62	1.10
	20–40	9.00	6.00	290.00	0.26	8.00	5.75	1.34	1.10
	40–110	7.00	6.00	150.00	0.26	8.00	5.75	1.34	0.99
SYS15	0–20	11.00	16.00	360.00	1.06	9.00	19.44	1.34	1.97
	20–40	10.00	7.00	270.00	1.21	7.00	26.30	1.22	1.09
	40–90	6.00	7.00	140.00	0.22	7.00	11.80	1.28	1.08
SYS16	0–20	11.00	13.00	370.00	0.69	9.00	11.00	1.80	1.08
	20–40	10.00	6.00	280.00	0.12	7.00	9.00	1.72	0.79
	40–100	5.00	5.00	160.00	0.13	7.00	8.40	2.15	1.08
SYS17	0–20	10.00	13.00	380.00	0.19	10.00	5.90	1.84	1.08
	20–50	9.00	5.00	290.00	0.25	6.00	5.70	1.72	1.05
	50–120	7.00	5.00	180.00	0.15	6.00	7.00	2.15	1.08
SYD1	20–0	0.07	14.49	540.62	1.81	14.18	27.93	1.28	0.52
	60–20	0.04	10.18	423.29	1.36	13.47	24.31	0.74	0.45
	100–60	0.02	4.09	119.37	1.31	16.92	24.85	0.69	0.49
SYD2	20–0	0.07	16.26	613.49	1.72	13.84	22.67	1.29	0.63
	60–20	0.03	10.87	495.83	1.49	15.53	20.12	0.93	0.55
	100–60	0.02	3.81	135.67	0.96	15.71	19.96	0.89	0.59
SYD3	15–0	0.06	16.89	477.20	2.10	15.21	23.93	1.18	0.58
	60–15	0.03	11.37	253.32	1.31	14.45	21.19	0.89	0.42
	110–60	0.01	3.14	81.12	0.90	15.88	17.39	0.80	0.55
SYD4	18–0	0.07	17.35	489.74	2.28	13.90	21.65	1.22	0.39
	60–18	0.03	9.15	320.91	1.36	14.58	18.23	0.91	0.42
	110–60	0.01	3.97	96.48	0.91	16.34	16.87	0.97	0.42
SYD5	20–0	0.07	15.66	488.49	1.74	15.67	27.36	1.31	0.54
	60–20	0.03	6.81	213.83	0.92	14.52	23.12	0.86	0.59
SYD6	20–0	0.07	14.20	456.38	2.19	14.42	25.37	1.39	0.43
	55–20	0.04	4.38	326.80	1.05	13.67	23.48	0.87	0.40
	80–55	0.02	2.17	107.18	0.69	17.94	18.26	0.66	0.62
SYD7	0–12	0.07	12.34	451.08	1.40	12.14	19.97	1.19	0.52
	40–12	0.04	7.27	327.59	1.16	11.59	17.85	0.70	0.41
	85–40	0.01	2.18	186.19	1.11	14.92	16.47	0.53	0.46
	130–85	0.01	1.22	42.95	0.91	14.38	16.19	0.40	0.25
SYD8	0–10	0.07	10.17	498.66	1.23	11.16	18.31	0.91	0.38
	38–10	0.03	4.21	313.09	1.11	11.27	18.06	0.70	0.57
	80–38	0.01	2.83	102.53	1.11	12.66	15.20	0.65	0.59
	125–80	0.01	1.78	39.64	0.87	12.91	11.73	0.48	0.24
SYD9	30–0	0.06	11.86	548.21	1.59	10.32	18.89	0.77	0.53
	60–30	0.02	3.61	267.13	1.33	11.48	15.22	0.43	0.39
	100–60	0.01	1.18	81.63	0.86	13.35	10.54	0.45	0.37
	150–100	–	–	70.29	0.60	13.59	8.58	0.31	0.12
SYD10	22–0	0.09	10.57	496.73	1.15	10.93	17.58	0.72	0.59
	55–22	0.03	3.68	411.54	0.93	10.34	16.69	0.54	0.66
	100–55	0.01	1.92	106.91	0.67	11.27	12.35	0.35	0.63
	140–100	0.01	1.13	102.77	0.40	12.96	10.38	0.24	0.19
SYD11	25–0	0.52	9.63	441.92	1.10	10.35	13.15	0.77	0.58
	50–25	0.02	3.12	378.07	1.23	11.81	12.34	0.59	0.55
	100–50	0.01	1.27	134.92	0.79	11.88	8.97	0.53	0.60
	130–100	–	–	111.68	0.36	13.37	8.24	0.37	0.23
SYD12	20–0	0.05	11.51	518.62	1.26	11.18	13.07	0.71	0.40
	85–20	0.03	4.10	274.10	1.15	14.38	10.56	0.56	0.48
	110–85	0.01	1.21	213.29	0.55	14.91	9.98	0.49	0.42
SYD13	0–10	0.05	13.14	538.55	1.09	10.68	12.37	0.75	0.49
	55–10	0.04	3.46	403.80	0.95	12.93	11.19	0.59	0.21
	55–120	0.01	2.65	216.26	0.64	12.52	8.56	0.38	0.28
SYD14	25–0	0.07	12.81	537.14	1.27	9.78	12.18	0.93	0.57
	70–25	0.03	3.23	398.59	1.14	12.32	9.25	0.54	0.46
	120–70	0.01	1.42	247.19	0.69	13.55	8.19	0.40	0.41
SYD15	0–10	0.06	12.49	611.27	1.31	11.92	11.43	0.71	0.65
	50–10	0.03	5.53	465.01	1.37	11.28	9.32	0.63	0.51
	100–50	0.01	1.28	193.75	0.85	14.30	9.78	0.41	0.30
SYD16	10–0	0.06	11.89	623.74	1.14	11.75	12.33	0.77	0.59
	45–10	0.04	6.67	470.22	1.23	11.26	10.12	0.49	0.67
	70–45	0.01	2.19	269.28	0.68	13.42	9.24	0.53	0.55
	120–70	0.01	0.54	113.97	0.53	13.75	7.39	0.27	0.24
SYD17	0–12	0.07	10.21	583.94	1.20	11.37	11.71	0.87	0.67
	50–12	0.04	4.38	416.30	1.06	10.22	9.67	0.56	0.48
	80–50	0.02	2.21	197.80	0.69	11.60	10.13	0.42	0.53
	135–80	0.01	0.87	185.66	0.44	13.76	7.62	0.29	0.18
SYD18	0–10	0.05	9.23	641.37	1.85	12.13	11.96	0.64	0.73
	40–10	0.03	7.82	429.82	0.90	12.97	10.37	0.51	0.48
	110–40	0.01	2.12	326.15	0.54	14.82	8.28	0.34	0.23
SYD19	30–0	0.08	7.39	491.82	1.43	11.43	10.62	0.88	0.58
	90–30	0.02	3.84	277.19	1.19	13.35	10.29	0.65	0.40
	140–90	0.01	1.67	101.54	0.91	14.98	9.16	0.57	0.38
SYD20	0–10	0.06	10.30	649.32	1.16	10.98	11.23	0.93	0.41
	50–10	0.03	3.65	405.39	0.92	11.93	9.59	0.66	0.48
	110–50	0.01	1.14	221.94	0.82	13.74	10.21	0.75	0.45
SYD21	0–12	0.06	8.21	579.08	1.38	10.91	10.44	0.61	0.24
	50–12	0.04	5.94	358.64	0.97	12.32	9.12	0.50	0.29
	120–50	0.01	1.19	189.24	0.51	13.49	10.26	0.36	0.33
SYD22	0–12	0.06	10.94	525.81	1.17	11.91	12.65	0.93	0.56
	60- 12	0.03	4.78	513.21	1.29	11.75	10.16	0.54	0.41
	90 −60	0.01	2.18	295.34	1.20	11.37	10.36	0.33	0.53
	130- 90	0.01	1.25	167.61	0.87	12.45	9.64	0.33	0.62
SYD23	32–0	0.07	9.49	479.65	1.25	11.83	13.16	0.71	0.61
	75–32	0.03	3.14	396.34	1.12	11.17	10.67	0.78	0.52
	120–75	0.01	1.27	114.07	0.88	13.48	9.34	0.64	0.47
SYD24	30–0	0.11	8.59	515.10	1.02	9.21	11.33	0.91	0.59
	65–30	0.08	3.80	318.28	1.15	12.65	9.48	0.54	0.51
	110–65	0.02	2.13	144.37	0.95	14.94	9.07	0.38	0.49
SYD25	28–0	0.10	9.37	542.93	1.19	11.39	11.78	0.82	0.51
	60–28	0.03	4.72	411.80	1.19	12.47	9.27	0.61	0.70
	110–60	0.02	1.91	219.23	0.84	12.13	10.17	0.35	0.46
SYD26	10–00	0.06	10.64	603.31	1.13	9.82	11.23	1.32	0.49
	50–10	0.03	4.30	486.39	0.81	10.77	9.53	0.57	0.38
	110–50	0.01	1.26	254.74	0.63	13.19	7.94	0.41	0.41
SYD27	20–0	0.05	8.83	493.19	1.22	10.54	12.49	1.10	0.65
	50–20	0.03	6.51	315.67	1.11	10.85	9.27	0.83	0.61
	100–50	0.01	1.14	195.87	0.60	12.31	8.79	0.53	0.34
	150–100	0.01	0.72	127.17	0.62	12.17	6.13	0.36	0.24
SYD28	18–0	0.05	9.48	459.91	1.14	11.68	10.41	0.86	0.60
	50–18	0.02	5.92	436.04	1.25	10.81	9.66	0.73	0.42
	80–50	0.01	1.13	216.74	0.82	12.89	9.32	0.51	0.29
	140–80	0.01	0.81	172.59	0.73	12.22	6.18	0.23	0.13
SYD29	25–0	0.05	10.67	469.30	1.17	9.31	10.10	0.75	0.59
	60–25	0.02	2.98	218.79	1.23	9.27	7.32	0.68	0.42
	90–60	0.01	1.14	141.16	1.12	11.63	7.14	0.61	0.44
	130–90	–	–	132.29	0.79	11.29	6.56	0.39	0.28
SYD30	22–0	0.05	11.93	486.10	1.24	11.27	10.63	0.57	0.57
	60–22	0.03	2.67	362.95	0.89	13.68	8.18	0.50	0.29
	100–60	0.01	1.97	196.60	0.75	13.38	7.23	0.26	0.20
SYD31	33 –0	0.06	10.42	527.68	1.13	11.28	9.31	0.64	0.66
	105–33	0.02	5.35	309.81	1.19	12.57	9.39	0.52	0.63
	160–105	0.01	1.18	145.34	0.59	14.25	7.14	0.36	0.31
SYD32	20–0	0.04	6.94	382.62	1.29	9.87	9.31	0.55	0.67
	50–20	0.02	5.82	350.22	76.00	10.25	6.20	0.51	0.45
	100–50	0.01	1.57	196.51	0.70	12.67	4.86	0.29	0.45
	140–100	–	–	120.48	0.53	10.81	4.16	0.26	0.21
SYD33	30–0	0.04	8.97	390.25	0.95	9.17	9.40	0.64	0.16
	60–30	0.02	2.62	252.95	1.14	11.28	6.58	0.67	0.22
	100–60	0.01	1.23	178.91	0.89	12.12	6.17	0.22	0.24
	140–100	–	–	138.63	0.54	11.69	4.34	0.18	0.27
SYD34	18–0	0.03	7.15	410.08	1.10	7.12	8.91	0.60	0.54
	70–18	0.02	2.87	231.95	1.17	10.44	4.17	0.49	0.55
	130–70	0.01	0.93	158.49	0.72	11.73	3.22	0.48	0.40
SYD35	30–0	0.04	6.48	395.42	1.21	9.13	9.62	0.63	0.61
	100–30	0.02	3.82	187.95	0.64	11.61	4.34	0.68	0.41
	160–100	0.01	0.78	101.33	0.38	11.96	3.47	0.36	0.33
SYD36	20–0	0.04	6.87	213.52	0.93	2.14	2.67	0.44	0.47
	20–70	0.02	2.79	203.45	0.84	4.23	1.92	0.39	0.30
SYD37	20–0	0.02	3.81	191.79	0.82	4.78	2.39	0.41	0.23
	20+	–	–	–	–	–	–	–	–
SYD38	30–0	0.03	5.67	313.40	0.89	2.19	3.70	0.54	0.18
	80–30	0.01	4.48	204.97	0.61	4.58	3.18	0.21	0.23
	120–80	0.01	1.17	159.22	0.38	5.97	1.42	0.21	0.12
SYD39	20–0	0.03	4.81	259.11	0.76	3.81	4.24	0.46	0.42
	50–20	0.01	4.28	219.36	0.81	7.24	3.79	0.30	0.32
	110–50	0.01	1.34	208.19	0.67	6.86	1.61	0.21	0.35
SYD40	30–0	0.03	5.31	207.45	0.71	3.45	6.25	0.49	0.34
	60–30	0.02	3.46	154.63	0.83	3.18	5.82	0.55	0.31
	100–60	0.01	0.78	86.90	0.65	6.32	3.26	0.35	0.29
	140–100	0.01	0.77	60.35	0.48	4.87	3.16	0.29	0.14
SYD41	0–12	0.02	4.15	209.13	0.80	3.91	1.88	0.45	0.26
	12 <	–	–	–	–	–	–	–	–
SYD42	20–0	0.03	2.41	240.01	0.91	2.16	4.70	0.39	0.33
	50–20	0.02	1.76	227.18	0.59	2.98	2.34	0.26	0.20
	80–50	0.01	0.32	166.21	0.45	4.47	1.86	0.25	0.26
	120–80	–	–	101.67	0.39	5.13	1.57	0.28	0.15
SYD43	25–0	0.02	2.56	216.57	0.81	2.71	1.19	0.66	0.41
	25 <	–	–	–	–	–	–	–	–
SYD44	15–0	0.02	3.18	381.67	0.92	2.67	2.52	0.61	0.29
	40–15	0.01	1.95	238.90	0.72	3.20	2.11	0.40	0.26
	80–40	0.01	1.20	133.64	0.65	3.99	1.28	0.27	0.22
	120–80	–	–	126.34	0.51	3.87	1.18	0.24	0.18
SYD45	25–0	0.03	1.59	205.92	0.71	3.25	1.48	0.47	0.24
	< 25	———-							
SYD46	20–0	0.03	2.26	276.59	0.98	2.59	3.93	0.45	0.38
	75–20	0.02	1.82	113.37	0.80	2.74	3.85	0.51	0.36
	115–75	0.01	0.72	95.98	0.57	4.80	2.27	0.33	0.27
	140–115	0.01	0.68	42.38	0.44	3.25	2.11	0.28	0.17
SYD47	25–0	0.03	3.87	238.89	0.82	3.42	4.21	0.49	0.26
	65–25	0.01	1.11	163.47	0.88	5.37	2.68	0.41	0.27
	100–65	0.01	0.61	107.49	0.59	5.10	2.33	0.38	0.24

**Table 5:** Cadmium (Cd) and Led (Pb) concentrations in some representative soil profiles in the southern region of Syria.

Profile	Horizon (cm)	Cd mg/kg	Pb
SYSW1	18–0	0.132	0.084
	40–18	0.134	0.039
	100–40	–	–
SYSW2	22–0	0.145	0.10
	45–22	0.115	0.09
	75–45	–	–
SYSW3	25–0	0.13	0.103
	90–25	0.127	0.068
SYSW4	22–0	0.096	0.201
	50–22	0.069	0.09
	85–50	–	–
SYSW5	12–0	0.141	0.13
	50–12	0.12	0.08
SYSW6	18–0	0.139	0.144
	60–18	0.128	0.076
	90–60	–	–
SYSW7	17–0	0.133	0.124
	30–17	0.119	0.122
	50–30	–	–
	110–50	–	–
SYSW8	12–0	0.12	0.044
	50–12	0.121	0.04
SYSW9	22–0	0.105	0.041
	35–22	0.096	0.022
	60–35	–	–
SYSW10	25–0	0.131	0.069
	40–25	0.116	0.06
	90–40	–	–
SYSW11	25–0	0.134	0.215
	80–25	0.138	0.109
	100–80	–	–
SYSW12	18–0	0.177	0.136
	55–18	0.163	0.079
	120–55	–	–
SYSW13	20–0	0.131	0.111
	45–20	0.127	0.057
	70–45	–	–
SYSW14	18–0	0.126	0.145
	40–18	0.128	0.088
	65–40	–	–
	100–65	–	–
SYSW15	18–0	0.202	0.116
	50–18	0.147	0.098
	90–50	–	–
SYSW16	18–0	0.142	0.174
	35–18	0.14	0.110
	70–35	–	–
	100–70	–	–
SYSW17	25–0	0.109	0.098
	50–25	0.110	0.070
	80–50	–	–
SYSW18	15–0	0.111	0.07
	40–15	0.108	0.06
	85–40	–	–
	110–85	–	–
SYSW19	20–0	0.124	0.151
	50–20	0.128	0.101
	100–50	–	–
SYSW20	18–0	0.11	0.035
	50–18	0.103	0.03
	110–50	–	–
SYSW21	14–0	0.13	0.133
	50–14	0.127	0.074
	70–50	–	–
	95–70	–	–
SYSW22	15–0	0.099	0.221
	60–15	0.087	0.142
	110–60	–	–
SYSW23	22–0	0.127	0.068
	60–22	0.125	0.055
	95–60	–	–
SYSW24	0–15	0.104	0.187
	15–60	0.099	0.141
	60–110	–	–
SYSW25	0–20	0.213	0.164
	20–50	0.162	0.09
	50–100	–	–
SYSW26	16–0	0.117	0.104
	40–16	0.121	0.044
	80–40	–	–
	100–80	–	–
SYSW27	0–10	0.231	0.177
	10–50	0.155	0.101
	90–50	–	–
SYSW28	18–0	0.081	0.239
	90–18	0.071	0.078
	120–90	–	–
SYSW29	15–0	0.099	0.242
	50–15	0.091	0.188
	130–50	–	–
SYSW30	20–0	0.095	0.077
	55–20	0.097	0.078
	100–55	–	–
SYSW31	15–0	0.088	0.117
	60–15	0.082	0.07
	110–60	–	–
SYSW32	0–10	0.088	0.198
	30–10	0.097	0.127
	60–30	–	–
	120–60	–	–
SYSW33	18–0	0.119	0.104
	50–18	0.104	0.054
	90–50	–	–
	100–90	–	–
SYSW34	18–0	0.081	0.146
	50–18	0.086	0.084
	80–50	–	–
	150–80	–	–
SYSW35	25–0	0.129	0.087
	50–25	0.123	0.065
	90–50	–	–
	180–90	–	–
	220–180	–	–
SYSW36	15–0	0.094	0.32
	50–15	0.086	0.199
	100–50	–	–
SYSW37	20–0	0.082	0.125
	60–20	0.082	0.052
	120–60	–	–
SYSW38	18–0	0.087	0.068
	50–18	0.079	0.059
	90–50	–	–
	130–90	–	–
	200–130	–	–
	260–200	–	–

## 2 Experimental design, materials, and methods

A total of 107 representative soil profiles were selected to represent the different agroecosystems in the southern part of Syria. The representative soil profiles were excavated until the rock parent materials. All soil profiles were fully described in the field following the FAO guidelines [1]. A total of 308 soil samples were collected from different soil horizons and analyzed for physicochemical and mineralogical properties in the laboratory. Soil samples were fractionated for sand, silt, and clay using the hydrometer method [2] and the percentage of the fractions were used to determined the soil texture type using the USDA particle size classification [3]. The cylinder and pycnometer methods were used to determine the soil bulk and particle densities, respectively [4]. Soil pH was measured in a 1:2.5 (soil:water) ratio using a digital pH meter (GH Zeal Ltd., Mi150, UK) as given in [5]. The soil electrical conductivity (EC) was measured in a 1:5 extraction using a digital EC meter (GH Zeal Ltd., Mi170, UK) according to [6]. Exchangeable Ca^+2^ and Mg^+2^ were determined using the titration method, while exchangeable Na^+^ and *K*^+^ were measured using a flame photometer (Microprocessor 1382), all extracted with 1 M NH_4_OAc (pH = 7.0) according to [7,8]. Total calcium carbonate equivalent (CCE) was determined using the calcimeter method [9]. Total organic carbon (TOC) was determined using the wet digestion method [10]. Cation exchange capacity (CEC) was determined following the 1 M NH_4_OAc (pH = 7.0) extraction method [11]. Total N was determined using Kjeldahl distillation method [12]. Olsen method [13] was followed to determine the available phosphorus (P) using spectrophotometer, whereas available K was determined in a 1:5 (soil: 1 M NH_4_OAc) using flame photometer according to [14]. Available Fe, Cu, Mn, and Zn were extracted with a DTPA extraction (pH = 7.3) method [15] using atomic absorption spectrophotometer (AAS-Jones, 2001). Available Boron was extracted with an acid hot water (0.05 M HCl) and determined followed colorimetric method using Azotomethan-H [16]. Cadmium (Cd) and lead (Pb) were determined in a DTPA extraction [15] using the AAS instrument (Jones, 2001). Mineralogy for powder and clay samples was determined using an X-ray diffractometer (MAXima_X XRD-7000, Shimadzu, Japan) link to PC-APD diffraction software at the Nuclear Energy Authority Laboratories (NEALs), Damascus, Syria. The XRD patterns were interpreted using the guidelines given by [17]. The soils were classified using the US *Soil Taxonomy* [18].

## Declaration of Competing Interest

The authors declare that they have no known competing financial interests or personal relationship that could have appeared to influence the work reported in this paper.
